# A Novel Monoclonal Antibody Against a Modified Vaccinia Ankara (MVA) Envelope Protein as a Tool for MVA Virus Titration by Flow Cytometry

**DOI:** 10.3390/v16101628

**Published:** 2024-10-17

**Authors:** Simeon Cua, Brenda A. Tello, Mafalda A. Farelo, Esther Rodriguez, Gabriela M. Escalante, Lorraine Z. Mutsvunguma, Javier Gordon Ogembo, Ivana G. Reidel

**Affiliations:** 1 Department of Immuno-Oncology, Beckman Research Institute of City of Hope, Duarte, CA 91010, USA; 2 Irell & Manella Graduate School of Biological Sciences, City of Hope, Duarte, CA 91010, USA

**Keywords:** modified vaccinia Ankara, virus titration, plaque-forming unit assay, flow cytometry, TCID_50_, monoclonal antibody, cell surface binding protein, OPG105, MVA105L, D8L

## Abstract

Modified vaccinia Ankara (MVA) virus is a widely used vaccine platform, making accurate titration essential for vaccination studies. However, the current plaque forming unit (PFU) assay, the standard for MVA titration, is prone to observer bias and other limitations that affect accuracy and precision. To address these challenges, we developed a new flow cytometry-based quantification method using a highly specific monoclonal antibody (mAb) for the detection of MVA-infected cells, as a more accurate titration assay. Through previous work, we serendipitously identified three MVA-specific hybridoma antibody clones, which we characterized through ELISA, immunoblot, and flow cytometry, confirming their specificity for MVA. Sequencing confirmed that each antibody was monoclonal, and mass spectrometry results revealed that all mAbs target the MVA cell surface binding protein (CSBP, MVA105L). We next optimized the titration protocol using the most effective mAb, 33C7 by refining culture conditions and staining protocols to enhance sensitivity and minimize background. Our optimized method demonstrated superior sensitivity, reliability, and reduced processing time when compared with the traditional PFU assay, establishing it as a more accurate and efficient approach for MVA titration.

## 1. Introduction

Modified vaccinia Ankara (MVA)—family Poxviridae, genus Orthopoxvirus, Species vaccinia virus—is a highly attenuated vaccinia strain that was generated after extensive passaging (~500 passages) of the Chorioallantois vaccinia Ankara virus strain (VACV) in chicken embryo fibroblast (CEF) cells [1,2,3]. Due to its large capacity for transgene insertion, endogenous immunogenic properties, and excellent human safety profile (arising from its limited cell tropism), MVA has become a widely used vaccine platform [4]. It has been successfully tested in early and late-stage vaccine clinical trials against multiple pathogens, including Ebola [5], human immunodeficiency virus [6,7,8], cytomegalovirus [9,10], and coronavirus [11], and is currently licensed for use against smallpox and monkeypox [4,12]. For any pre-clinical and clinical MVA-based vaccine studies, the accurate measurement of virus titer in a timely manner is critical [13].

Currently, there are two standard methods for quantifying MVA titer: the plaque forming unit (PFU) assay and the median tissue culture infectious dose (TCID_50_) endpoint assay [13,14]. In both assays, serially diluted virus is used to infect a monolayer of adherent cells followed by a direct observation of localized areas for cytopathic effects (CPE) after staining. Unlike vaccinia virus, MVA does not form distinct plaques in producer cells lines and therefore requires staining for visualization, which is a multi-step and laborious process [13]. Additionally, the accuracy and precision of the assays can be hampered by observer bias in manual counting, and by the potential emergence of secondary infections depending on experimental timing. An alternative approach measures either viral gene or transgene expression in infected cells via flow cytometry [15,16]. This approach eliminates observer bias, and also offers a greater potential for high-throughput processing. Furthermore, the expression of vaccine antigens can be detected simultaneously, and data measurements can be based on a higher number of infectious events and experimental replicates.

In a recent study, Li et al. optimized a method for flow cytometry-based MVA quantification using an anti-vaccinia polyclonal antibody in BHK-21 cells [16]. Results using this method were comparable to those obtained using standard TCID_50_ assay measurements in CEF cells. To improve upon these existing methods and to standardize a general flow cytometry-based method for MVA quantification, we have validated a new MVA-specific monoclonal antibody (mAb) as a specific reagent for use in flow cytometry-based MVA titer quantification. We serendipitously generated this mAb after using recombinant transgene-expressing MVA as an immunogen for hybridoma generation against a human herpesvirus 4 protein (HHVp), and, after characterizing its functionality in ELISA, immunoblot, and flow cytometry assays, we also identified its binding target via mass spectrometry analysis. We then used this mAb to optimize flow cytometry staining conditions and additional critical assay parameters for MVA titer quantification. As a proof-of-concept, we used our optimized protocol to quantify the viral titer of two recombinant MVA vectors and systematically compared the results from this assay to those obtained using the standard PFU assay. Our new mAb, incorporated into the optimized protocol, outperformed the PFU assay, providing a powerful and innovative tool for MVA research.

## 2. Materials and Methods

### 2.1. Animals, Cell Lines, and Viruses

A female BALB/c mouse (aged 8–10 weeks) was purchased from Charles River Laboratories (Strain #028) and used for antibody generation. The mouse was housed at BSL2 facilities in the Animal Resource Center of Beckman Research Institute of City of Hope, with free access to food and water in a 12:12 light/dark cycle. The animal facilities are accredited by the Association for Assessment and Accreditation of Laboratory Animal Care (AAALAC) and conform to the Institute for Laboratory Animal Research Guide for the Care and Use of Laboratory Animals. All mouse procedures were performed in accordance with approved Beckman Research Institute of City of Hope Institutional Biosafety Committee (IBC, #16001) and Institutional Animal Care and Use Committee (IACUC, #16003) protocols.

Baby hamster kidney cells 21 (BHK-21; CCL-10 C-13) and human embryonic kidney cells, HEK-293 (CRL-1573) and HEK-293T (CRL-3216), were purchased from ATCC and grown in Dulbecco’s Modified Eagle Medium (DMEM; 10-013-CM, Corning, Corning, NY, USA). Chicken embryo fibroblasts (CEF; 10100795, Charles River Avian Vaccine Services, now AVS Bio, Norwich, CT, USA) were grown in Minimum Essential Medium (MEM; 25-504, GenClone, Morrisville, NC, USA). All the cell culture media were supplemented with 10% fetal bovine serum (FBS; 25-550, GenClone), 2% Gibco™ penicillin-streptomycin (15140122, ThermoFisher Scientific, Waltham, MA, USA), and 1% Gibco™ L-glutamine (25030081, ThermoFisher Scientific), unless otherwise noted. Cells were cultured at 37 °C in a 5% CO_2_ humidified incubator. ExpiCHO-S cells were obtained as part of the Gibco™ ExpiCHO^TM^ Expression System (A29133, ThermoFisher Scientific) and maintained as per the manufacturer’s instructions.

MVA-BAC-TK (in this article referred to as MVA-eGFP) harbors the MVA genome with the BAC pBeloBAC11 (GenBank: U51113) inserted at the position of the TK gene together with an eGFP expression cassette. The MVA-BAC-TK in GS1783 bacteria was a kind gift from Dr. Don Diamond (Beckman Research Institute of City of Hope, Duarte, CA, USA), and was developed from the MVA 1974/NIH clone 1, obtained under MTA from Dr. Bernard Moss (National Institutes of Allergy and Infectious Diseases, Bethesda, MD, USA). The MVA-BAC-TK has been previously described [17], as has GS1783 bacteria [18]. Two recombinant MVA viruses expressing a human herpesvirus protein (HHVp) with and without a 6x histidine tag (His), MVA-HHVp-His and MVA-HHVp, respectively, were generated in our laboratory via homologous recombination techniques using MVA-BAC-TK in GS1783 bacteria [18], and reconstituted in BHK-21 as described by Chiuppesi and Nguyen et al. [19]. Due to its association with other projects currently in development in our lab, we are abstaining from disclosing the identity of the HHVp in this current article. An MVA virus encoding for five EBV glycoproteins, gp350, gB, and the complex gp42gHgL reported by Escalante and Reidel et al. [20], which had the pBeloBAC11 and eGFP cassettes removed (MVA-EBV5-2 A5-1), was also generated in our laboratory using the previously mentioned technology.

### 2.2. Large-Scale Production of MVA Viruses

All viruses were grown and expanded in BHK-21 cells for large-scale production and purified via ultra-centrifugation using a sucrose cushion, as previously described [13,21]. Briefly, the BHK-21 cells were expanded to 70–100 15 cm dishes. After reaching 80–90% confluency, the culture media was removed, and the cells were incubated for 1 h with virus diluted in DMEM without supplements, followed by the addition of complete DMEM for further cell culture. The cells were maintained and grown until achieving 90–100% infection based on eGFP expression or cytopathic effect evaluation for eGFP-positive and -negative viruses, respectively. The infection was tracked by visual inspection using an inverted microscope including a GFP fluorescence channel (Invitrogen™ EVOS™ FL Digital Inverted Fluorescence Microscope, AMF4300, ThermoFisher Scientific). To isolate the viruses, the cells were harvested using a cell scraper, then pelleted by centrifugation at 3724× *g* for 30 min at 4 °C (Allegra-X14R, Beckman Coulter, Brea, CA, USA), and the supernatant was discarded. The cell pellets were washed with 1X PBS following the same procedure. Subsequently, the cell pellets were frozen at −80 °C and thawed for three cycles, then resuspended in 10 mM Tris-Cl and homogenized using a glass Dounce homogenizer, followed by sonication (100 amplitude; Q500, Qsonica, Newtown, CT, USA) for 4 min. The resulted homogenate was loaded on a 36% sucrose cushion followed by ultracentrifugation at 43,700× *g* for 80 min at 4 °C (SW32 Ti rotor, Beckman Coulter). The cell pellet was resuspended in Gibco™ OptiMEM (31985070, ThermoFisher Scientific), aliquoted in cryovials and stored at −80 °C for titration experiments and immunizations.

### 2.3. HHVp-His Production and Purification

The BHK-21 cells were expanded to 70 15 cm tissue culture dishes. After reaching 80–90% confluency, the cells were infected with MVA-HHVp-His following the procedure described in the previous section. At 48 h post infection, the cells were harvested by scraping, pelleted by centrifugation at 3724× *g* for 30 min at 4 °C, and the supernatant was discarded. The cell pellet was washed with 1X PBS and lysed in xTractor buffer (635671, Takara Bio, San Jose, CA, USA) supplemented with Thermo Scientific™ protease inhibitor (A32953, ThermoFisher Scientific) using a 1 g:20 mL ratio (cell pellet:xTractor buffer). Lysate was clarified at 4000× *g* for 20 min at 4 °C and filtered through 0.45 µm PES membrane. Lysate was loaded on a HisTrap FF column (17528601, Cytiva, Marlborough, MA, USA) and the major elution peak was collected. The eluted protein was concentrated using a Vivaspin 20 column with a molecular weight cut-off (MWCO) of 10 kDA (28932360, Cytiva) and evaluated by SDS-PAGE, followed by Coomassie staining. This semi-purified HHVp was used for ELISA, immunoblot, and mass spectrometry, as described later in this article. For immunization purposes, HHVp was further purified by size exclusion chromatography. Briefly, the semi-purified HHVp was loaded on a HiLoad 16/600 Superdex^TM^ 200 pg column (28989335, Cytiva). Different fractions were collected based on chromatogram peaks at A280_nm_ and evaluated by SDS-PAGE and Coomassie staining analysis. In all cases, the HHVp’s final concentration was determined by A280_nm_ on a Thermo Scientific™ Nanodrop Lite Spectrophotometer (ThermoFisher Scientific).

### 2.4. Infection of BHK-21 and CEF Cells with MVA-eGFP and Lysis for Immunoblot Assessment

The BHK-21 and CEF cells were seeded in 15 cm dishes and T75 flasks, respectively. After reaching 80% confluency, the medium was replaced with 3 mL of MVA-eGFP diluted in basal DMEM or MEM. After 1 h incubation, an additional 17 mL of complete media was added. The cells were maintained and grown until achieving 90–100% infection based on eGFP expression. The cells were harvested using a cell scraper, pelleted at 4000× *g* for 5 min and then washed with PBS. The cell pellets were kept at −80 °C till further use in the immunoblot assessment. For this purpose, the cell pellets were lysed using GoldBio Mammalian lysis buffer (GB-180, GoldBio, St Louis, MO, USA) with protease inhibitor (11836170001, Roche, Indianapolis, IN, USA) according to the manufacturer’s recommendations and then clarified at 4000× *g* for 30 min. The supernatant recovered was kept at 4 °C for immediate use, or stored at −20 °C for later use. A similar procedure was followed in preparing uninfected BHK-21 and CEF cells used as controls in immunoblot.

### 2.5. Hybridoma Generation and Selection by ELISA and Immunoblot

**Figure 1:**
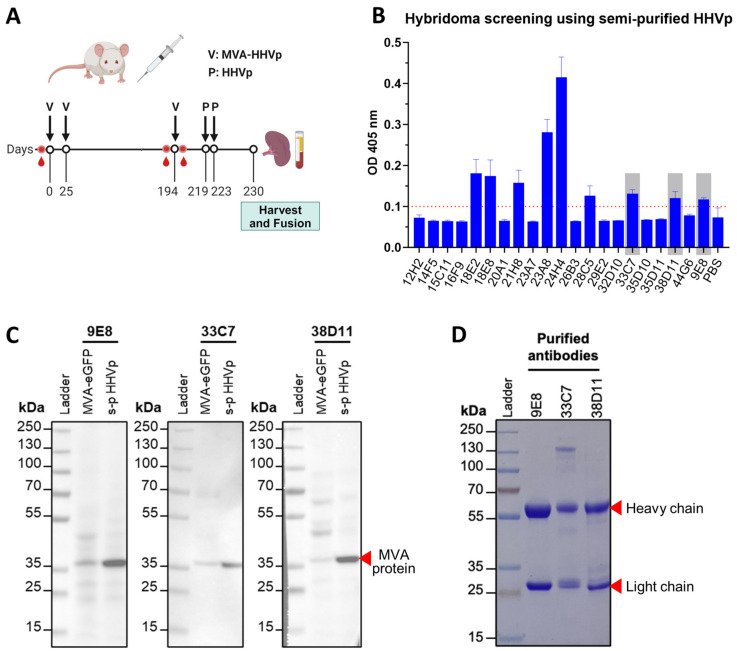
Generation and characterization of antibodies against modified vaccinia Ankara (MVA) virus. (**A**) Schematic diagram of the BALB/c mouse (*n* = 1) immunization schedule used to generate MVA/human herpesvirus protein (HHVp)-specific murine antibodies. Mouse was bled or immunized with either MVA expressing His-tagged MVA-HHVp (V) or purified His-tagged HHVp produced from cells infected with this virus (P) on the indicated days. Harvested spleen at the end of the study period was used to generate hybridomas via the myeloma cell fusion method. Created in BioRender. Cua, S. (2023) BioRender.com/o30l999 (**B**) Confirmatory ELISA screen of hybridoma supernatants to detect anti-HHVp antibodies. Culture supernatants from hybridomas generated in (**A**) were subjected to an initial HHVp-specific antibody ELISA screen. After expansion of the resulting ELISA-positive hybridoma clones, culture supernatants were further screened by a confirmatory ELISA (shown), using soluble semi-purified His-tagged HHVp produced in an MVA system as the target antigen. Each bar represents the mean OD_405nm_ of three replicates + the standard deviation; samples with a mean OD_405nm_ > 0.1 (dotted line) were considered positive. (**C**) Immunoblot assessment of hybridoma supernatants to detect anti-HHVp antibodies. Culture supernatants from hybridoma clones that were ELISA-positive in (**B**) were used as primary antibodies in immunoblot assay against lysates of MVA-eGFP-infected BHK-21 cells (MVA-eGFP) or semi-purified His-tagged HHVp (s-p HHVp) produced in an MVA system. Shown are the blots of hybridoma clones (3/9) that detected a protein band of the same size in both MVA-eGFP and s-p HHVp samples (indicated with red arrow), suspected to be an MVA-specific protein. Remaining blots (6/9), which did not detect this protein band, are shown in Figure A1B. (**D**) SDS-PAGE analysis of purified hybridoma supernatant antibodies. Antibodies from hybridoma supernatants shown in (**C**) were purified via protein A (9E8 and 33C7) or G (38D11) affinity chromatography and subjected to SDS-PAGE followed by Coomassie blue staining to assess antibody purity. Heavy and light chains are indicated with red arrows. See also Figure A1.

A female BALB/c mouse was immunized intraperitoneally three times with MVA-HHVp on days 0, 25, and 194 (Figure 1A), and one time with HHVp formulated with Freud’s incomplete adjuvant on day 219. On day 223, a final HHVp booster without adjuvant was applied via a tail vein injection and the mouse’s spleen was harvested on day 230. Splenocytes were isolated, purified, and fused with P3×63Ag8.653 cells (CRL-1580, ATCC, Manassas, VA, USA) at a 1:1 ratio using polyethylene glycol (P7181, Sigma-Aldrich, St. Louis, MO, USA)-mediated chemical fusion to generate hybridomas, as previously described in [22]. Resulting hybridoma supernatants were screened by indirect ELISA. Briefly, 96-well ELISA plates (514201, NEST, Woodbridge, NJ, USA) were coated overnight at 4 °C with 25 ng/well of semi-purified HHVp in PBS (50 µL at 0.5 µg/mL). Plates were blocked with 200 µL of 3% BSA (A9647, Sigma-Aldrich) 0.1% Tween-20 (P1379, Sigma-Aldrich) PBS (blocking buffer) for 1 h at room temperature, shaking. One hundred μL of hybridoma supernatant was added to each well in triplicates and incubated for 2 h at room temperature, shaking. The plates were then incubated for 1 h at room temperature, shaking, with 50 µL of HRP-conjugated anti-mouse IgG diluted 1/2000 in PBS (#1706516, Bio-Rad, Hercules, CA, USA). Three washes with 0.1% Tween in PBS were performed after each step. The plates were then incubated for 20 min with 100 µL of ABTS 2-Component Microwell Peroxidase Substrate (5120-0033, LGC SeraCare, Milford, MA, USA). The reactions were stopped using 100 µL of ABTS Peroxidase Stop Solution (5150-0017, LGC SeraCare), and the optical density (OD) of these reactions was read at 405 nm with a Filter Max F3 microplate reader (Molecular Devices, San Jose, CA, USA). All of the 21 ELISA-positive clones with an OD_405nm_ > 0.1 were expanded and kept in culture for an additional 2 weeks. The supernatants were harvested periodically and exchanged for fresh medium. The resulting pool of harvested supernatants for each clone was evaluated by a confirmatory ELISA, using the procedure described above. A total of 9 of the 21 clones were confirmed positive by ELISA (OD_405nm_ > 0.1) and further characterized by immunoblot using semi-purified HHVp and MVA-eGFP-infected BHK-21 lysate as targets. These protein samples were prepared for SDS-PAGE by boiling with an appropriate volume of 6x reducing Thermo Scientific™ Laemmli SDS sample buffer (J61337.AC, ThermoFisher Scientific) at 100 °C for 5 min, and loaded on an Invitrogen™ Bolt 4–12%, Bis-Tris Plus gel (NW04122BOX, ThermoFisher Scientific). After gel electrophoresis, the proteins were transferred to a nitrocellulose membrane via the Invitrogen™ iBlot 2 system (ThermoFisher Scientific), and subsequently blocked with blocking buffer for 1 h at room temperature, shaking. The membranes were then incubated overnight at 4 °C, shaking, with undiluted hybridoma supernatants. The membranes were washed three times with 0.1% Tween in PBS for 5 min, shaking. They were then incubated for 1 h at room temperature, shaking, with secondary HRP-conjugated anti-mouse (1706516, Bio-Rad) diluted 1/2000 in blocking buffer. The membranes were then washed as before and briefly incubated with Thermo Scientific™ SuperSignal West Pico PLUS Chemiluminescent Substrate (34578, ThermoFisher Scientific), after which they were immediately imaged in a PXi Multi-Application Gel Imaging System, Syngene, Bangalore, India. After antibody purification, a similar immunoblot procedure was followed using MVA-eGFP-the infected BHK-21 and CEF lysates as targets and the uninfected cells as negative controls. In this second immunoblot, after the development of the MVA-specific antibodies, blots were re-incubated with HRP-conjugated anti-beta actin (sc-47778, Santa Cruz Biotechnology, Dallas, TX, USA) diluted 1/1000 in blocking buffer and re-developed as a loading control. For the Coomassie stain analyses, protein gels after SDS-PAGE were placed in Coomassie Brilliant Blue staining solution (161-0436, Bio-Rad) for 1 h, shaking, and subsequently destained by incubation in destaining solution (10% acetic acid, 40% methanol in deionized water) for 12 h, shaking; resulting gels were imaged in a PXi Multi-Application Gel Imaging System. Antibody isotyping was done in three selected clones (9E8, 33C7 and 38D11) using the Thermo Scientific™ Pierce Rapid Isotyping Kit plus Kappa and Lambda—Mouse (26179, ThermoFisher Scientific) with 1:50–1:100 dilutions of hybridoma culture supernatant, according to the manufacturer’s instructions.

### 2.6. Antibody Purification

Selected antibody clones (9E8, 33C7, and 38D11) were purified in our laboratory by affinity chromatography using the AKTA Pure protein purification system (Cytiva). Clones 9E8 and 33C7 were purified using HiTrap Protein G columns (17040503, Cytiva) while clone 38D11 was purified using HiTrap Protein A columns (17040303, Cytiva), followed by preparative size exclusion chromatography. Briefly, hybridoma supernatants were clarified by centrifugation, filtered through 0.22 µm PES filter (SFPE3322-01, Bioland Scientific LLC), diluted at 1:1 *v*/*v* with Thermo Scientific™ Gentle Ab/Ag Binding buffer, pH 8.0 (21012, ThermoFisher Scientific) for Protein A columns, or Tris Buffered Saline, pH 7.4 (BP2472-1, Fisher BioReagents, Waltham, MA, USA) for Protein G columns. After loading the diluted supernatants into corresponding 5 mL affinity chromatography columns, the columns were washed with 25 mL equilibration buffer before a single step elution in 1 mL fractions using Thermo Scientific™ Gentle Ab/Ag Elution Buffer, pH 6.6 (21013, ThermoFisher Scientific). Elution peaks were monitored by A280_nm_ and resulting fractions were evaluated by SDS-PAGE followed by Coomassie staining, as described in the previous section. Antibody-enriched fractions were combined and buffer-exchanged to PBS using the Thermo Scientific™ Zeba Spin Desalting Column 7 kDa MWCO (89894, ThermoFisher Scientific), and concentrated using Vivaspin 20 columns with a 10 kDa MWCO. Concentrated fractions were loaded on a HiLoad 16/600 Superdex^TM^ 200 pg column (28989335, Cytiva) for size exclusion chromatography. Separated fractions were collected based on chromatogram peaks at A280_nm_ and evaluated by SDS-PAGE and Coomassie staining analysis, as described in the previous section. The final concentration was determined by A280_nm_ on a Nanodrop Lite Spectrophotometer. The binding capacity of the purified antibodies was confirmed by immunoblot, as described in the previous section, with the MVA-eGFP-infected BHK-21 and CEF lysates used as targets and the uninfected BHK-21 and CEF lysates as controls.

### 2.7. Assessment of Antibody Binding to Surface-Expressed Proteins in MVA-eGFP-Infected Cells by Flow Cytometry

BHK-21 and CEF cells were infected with MVA-eGFP, as described in Section 2.4. To achieve a single cell suspension, harvesting was performed using Gibco™ 0.25% trypsin/EDTA (25200-056, ThermoFisher Scientific). Briefly, the cell medium was removed, and the cells were washed once with PBS, followed by the addition of 2 mL 0.25% trypsin/EDTA per dish. After confirming cellular detachment, the reaction was stopped by the addition of 2 mL of staining buffer (2% FBS in PBS). Subsequently, the cell concentration was quantified and adjusted to 1 × 10^6^ cells/mL in staining buffer. The cell suspension was transferred to a 96-well V bottom plate (353263, Falcon, Corning, NY, USA) at 100 μL (1 × 10^5^ cells)/well for staining, and 100 μL of staining buffer was then added to each well, followed by a 15 min incubation at 4 °C and centrifugation at 500× *g* for 5 min. The cell pellets were resuspended in 50 μL of primary antibodies (9E8, 33C7 or 38D11) diluted at 30 μg/mL in staining buffer and incubated for 30 min at 4 °C. The secondary antibodies-only and unstained controls were resuspended with 50 μL of staining buffer with no primary antibodies. After incubation, the cells were washed two times by adding 250 μL/well of PBS followed by centrifugation at 500× *g* for 5 min and supernatant removal. Subsequently, the cells were resuspended in 50 μL of goat anti-Mouse IgG (H + L) Highly Cross-Adsorbed Secondary Antibody, Invitrogen™ Alexa Fluor™ Plus 647 (A32728, ThermoFisher Scientific) diluted 1/1000 in staining buffer and incubated for 30 min at 4 °C. Finally, the cells were washed, as previously described, and resuspended in 100 μL of 1% paraformaldehyde (PFA; 15710, Electron Microscopy Sciences, Hatfield, PA, USA) in PBS. The samples were kept in the dark at 4 °C for up to 48 h and then analyzed via flow cytometry in a NovoCyte Quanteon 4025 (Agilent, Santa Clara, CA, USA). Data analysis was performed using FlowJo v10.9 (BD Biosciences, Franklin Lakes, NJ, USA) and the results were expressed as the average of the quadruplicate sample measurements.

### 2.8. Antibody Sequencing and Recombinant Expression

CDR sequencing of the V_H_ and V_L_ chains of the selected anti-MVA antibodies was performed by GenScript, and the obtained sequences were subcloned into a mouse IgG1 and a mouse κ expression pcDNA 3.4 vector for the heavy and light chain, respectively. Recombinant antibody expression was done using the ExpiCHO^TM^ Expression System, according to the manufacturer’s Max Titer protocol. Briefly, the ExpiCHO-S cells were subcultured in 1 L flasks and seeded at 6 × 10^6^ cells/mL in 200 mL of ExpiCHO^TM^ expression medium on the day before transfection. Plasmid DNA (pDNA) was prepared by diluting 100 μg/each of heavy and light chain-encoding pDNA in 8 mL of OptiMEM and adding it to a mixture of 8 mL ExpiFectamine™ CHO Reagent in 37 mL of OptiMEM. The DNA-transfection reagent complex was added dropwise to cells while swirling the flask, and the cells were then incubated in a 37 °C incubator with a humidified atmosphere of 8% CO_2_, shaking (120 rpm). From 18 to 22 h post-transfection, 1.2 mL of ExpiFectamine™ CHO Enhancer and 32 mL of ExpiCHO™ Feed were added and the incubation conditions were modified to 32 °C with a humidified atmosphere of 5% CO_2_, shaking (120 rpm). On day 5, the cells were fed for a second time with 32 mL of ExpiCHO™ Feed. The cells were further cultured until day 13, when supernatant was harvested and spun for 1 h at 3700 g on a benchtop Allegra-X14R centrifuge (Beckman Coulter). The supernatant was transferred to a new tube and spun for an additional hour, followed by filtration through 0.22 µm PES membrane. Purification was done as previously described for hybridoma supernatant and the resulting antibodies were termed r9E8, r33C7, and r38D11. An additional batch of r33C7 was also expressed and purified by GenScript.

### 2.9. Specificity Evaluation of the Selected Anti-MVA Antibodies by Mass Spectrometry

To identify possible targets of the selected anti-MVA antibodies, a liquid chromatography mass spectrometry analysis (LC-MS) of the band recognized by immunoblot was performed by the Integrated Mass Spectrometry Shared Resource of City of Hope. Briefly, 5 µg of semi-purified HHVp were resolved by SDS-PAGE and stained with Coomassie Brilliant Blue, as described in Section 2.5. A band corresponding to the target band size identified by immunoblot was manually cut and destained with 25 mM ammonium bicarbonate in acetonitrile/ultrapure water (1:1). Next, the sample was reduced with 10 mM DTT at 60 °C for 30 min and alkylated in-gel with 55 mM iodoacetamide for 30 min at room temperature in the dark. The gel was dehydrated using acetonitrile and rehydrated with 20 ng/µL trypsin in 100 mM ammonium bicarbonate. The resulting gel pieces were incubated overnight at 37 °C. Digested peptides were extracted from the gel by treatment with 50 mM ammonium bicarbonate in 50% acetonitrile, 2.5% formic acid in 50% acetonitrile, and acetonitrile. Combined extracts were dried, resuspended in water with 2% acetonitrile and 0.1% formic acid, and desalted using Thermo Scientific™ C_18_ tips (PI84850, ThermoFisher Scientific). Peptides were analyzed by LC-MS on the Thermo Scientific™ Orbitrap Fusion™ Lumos™ Tribrid™ mass spectrometer (ThermoFisher Scientific) using the following conditions: 60 min LC gradient on 75 µm × 50 cm Thermo Scientific™ EasySpray column (ES803A, ThermoFisher Scientific), MS acquired in Orbitrap at 120,000 resolution and MS/MS acquired in ion trap using Higher-energy Collisional Dissociation (HCD) at 35% collision energy. Collected data was analyzed with Proteome Discoverer v2.4 running Byonic v3.7.13 against a combined database of MVA and Golden Hamster genome using a strict false discovery rate limit (1% at protein and peptide level). The search assumed fixed carbamidomethylation of cysteine, variable oxidation of methionine and deamidation of asparagine and glutamine, and tryptic cleavage with up to two missed cleavage sites.

### 2.10. Specificity Confirmation of Anti-MVA Antibodies by Binding Evaluation to Recombinant MVA Viral Proteins

Genes encoding for the mass spectrometry-identified proteins MVA cell surface-binding protein (CSBP, MVA105L) and IMV-heparin binding surface protein (IMV-HBP, MVA093L) were individually cloned with the addition of a His-tag into pTT3 vectors by GenScript. HEK-293T cells were seeded in 6-well tissue culture plates and transfected the following day with the plasmids, after confirming 70–80% cell confluency. Transfection was done using 1 to 2 µg of plasmid DNA per well complexed with polyethylenimine hydrochloride (PEI; 24765, Polysciences Inc., Warrington, PA, USA) in OptiMEM media at a DNA/PEI 1:4 (*w*:*w*) ratio. After 24 h, the cells were harvested and analyzed for immunoblot or flow cytometry using the 9E8, 33C7, and 38D11 anti-MVA antibodies, with transfected HEK-293T cells used as a target and the un-transfected HEK-293T cells as a negative control. For the immunoblot, the cells were harvested and processed as described in Section 2.4. As an immunoblot control to confirm CSBP and IMV-HBP recombinant expression, samples were additionally stained with an anti-His antibody (IH.8, sc-57958 Santa Cruz Biotechnology) at 1:1000–1:2000 dilutions in blocking buffer. For the flow cytometry evaluation, the cells were harvested and processed as described in Section 2.7.

### 2.11. Flow Cytometry Experimental Condition Optimization for MVA Viral Titer Measurement

To optimize the detection of MVA-infected cells by flow cytometry, two parameters were first assessed: the use of nocodazole to inhibit viral morphogenesis and subsequent secondary infection and the use of intracellular staining to increase the detection signal. The BHK-21 cells were seeded in 48-well tissue culture plates at 1 × 10^5^ cells/well density and grown overnight in a humidified incubator in 5% CO_2_ at 37 °C. On the following day, the medium was replaced with 100 µL of MVA-eGFP diluted in basal DMEM. After a 1 h incubation, the cells were washed two times with PBS and 500 µL/well complete DMEM with or without 0.2 µg/mL nocodazole was added, with two sets of triplicates per condition. The nocodazole concentration was selected based on results obtained after evaluating its effect on MVA-eGFP infectivity (see Figure A4). After a 20 h incubation, the cell medium was removed, and the cells were washed two times with PBS, followed by the addition of 100 µL 0.25% trypsin/EDTA per well. After confirming cellular detachment, the reaction was stopped by the addition of 100 µL of 10% FBS in PBS and the cells were transferred well to well into a 96-well V bottom plate for staining, followed by centrifugation at 500× *g* for 5 min. The cells were resuspended in 100 µL of Zombie NIR™ Fixable Viability dye (423105, BioLegend, San Diego, CA, USA) diluted 1/100 in staining buffer (PBS/0.5% BSA/2 mM EDTA), incubated 15 min at RT in the dark, and spun down at 500 g for 5 min. Subsequently, one set of triplicates was stained, as described in Section 2.7, with the secondary antibody replaced by a goat anti-Mouse IgG (H + L) Highly Cross-Adsorbed Secondary Antibody, Invitrogen™ Alexa Fluor™ Plus 555 conjugate (A32727, ThermoFisher Scientific) diluted 1/1000 in staining buffer. The second set of triplicates was processed for extra-/intra-cellular staining using the BD Cytofix/Cytoperm Fixation/Permeabilization Solution Kit (555028, BD Biosciences). Briefly, the cells were resuspended in 100 µL of BD Fixation/Permeabilization buffer and incubated 20 min at 4 °C, after which 100 µL of BD Perm/Wash buffer were added followed by centrifugation at 500× *g* for 5 min; cells were subsequently washed 2 times with 200 µL of BD Perm/Wash buffer. Cell pellets were then resuspended in primary antibody using 100 μL of anti-MVA 33C7 diluted at 30 μg/mL in staining buffer and incubated for 30 min at 4°C. Secondary-only and unstained controls were resuspended with 100 μL of BD Perm/Wash buffer with no primary antibody. After incubation, cells were washed two times by adding 200 μL/well of BD Perm/Wash followed by centrifugation at 500× *g* for 5 min. Subsequently, the cells were resuspended in 100 μL of goat anti-Mouse IgG (H + L) Highly Cross-Adsorbed Secondary Antibody, Alexa Fluor™ Plus 555 conjugate diluted 1/1000 in BD Perm/Wash buffer and incubated for 30 min at 4 °C. Finally, the cells were washed as previously described and resuspended in 100 μL of 1–1.5% PFA in PBS. After selecting the use of nocodazole and extra-/intra-cellular staining as optimal conditions for viral infection titration by flow cytometry, two additional optimizations were performed. First, the performance of 9E8, 33C7 and 38D11, was evaluated and compared by following the protocol described in this section with the use of these three anti-MVA antibodies as primary antibody, to identify the optimal antibody for subsequent assays. Second, the optimal concentration of the selected antibody (33C7) was determined by titration, using a 1/10 seven-point serial dilution starting at 30 μg/mL of 33C7. Finally, to confirm 33C7 binding to MVA-infected cells occurred only upon infection, this protocol was repeated using MVA-eGFP- and UV-treated (inactivated) MVA-eGFP-infected BHK-21 cells. In all cases, the flow cytometry samples were kept in the dark at 4 °C for up to 48 h before data collection and the data was collected in a NovoCyte Quanteon 4025 (Agilent). Data analysis was performed using FlowJo v10.9 and the results were expressed as the average of triplicate samples.

### 2.12. MVA Titer Determination by PFU and TCID_50_ Assays

PFU and TCID_50_ assays were performed in BHK-21 cells as previously described [13,14] with some modifications. For PFU, the cells were seeded in 6-well tissue culture plates at 6 × 10^5^ BHK-21 cells/well, while for TCID_50_, the cells were seeded in 96-well tissue culture plates at 2 × 10^4^ BHK-21 cells/well. After 24 h post seeding, the media was removed, and the cells were infected with 10-fold serial dilutions of the virus (ranging from 1/10 to 1/10^11^) in OptiMEM by adding 1 mL inoculum to each well of 6-well plates in duplicates, or 100 µL to each well of 96-well plates in 8 replicates. The plates were then incubated for 1 h in a humidified incubator at 37 °C with 5% CO_2_. After incubation, the virus-containing media was removed, and the cells were washed two times with PBS. Subsequently, 1 mL of complete culture media was added to the 6-well plates and 100 µL to the 96-well plates. The cells were then incubated overnight. After 24 h incubation, media was removed from wells and cells were washed with PBS before fixation in acetone/methanol (1:1) for 2 min. The cells were washed and stored in PBS at 4 °C until immunostaining was performed. Staining was performed using the VECTASTAIN^®^ Elite^®^ ABC-HRP Kit, Peroxidase (Sheep IgG) kit (PK-6106, Vector Laboratories, Newark, CA, USA), and DAB Substrate kit, Peroxidase (HRP) (SK-4100, Vector Laboratories), according to the manufacturer’s instructions with primary anti-vaccinia polyclonal antibody (9503-2507, Bio-Rad) diluted 1/2000 in PBS. Alternatively, the VECTASTAIN^®^ ABC-HRP Kit, Peroxidase (Mouse IgG) kit (PK-4002, Vector Laboratories) was used with purified hybridoma 33C7 or r33C7 (1 µg/mL) as the primary antibodies to complete the staining process. The plaque identification and counting was done using an EVOS™ FL Digital Inverted Fluorescence Microscope. Infectious units per mL (IU/mL) for the PFU assay were calculated by multiplying the average plaque count between duplicates by the dilution factor and dividing by the inoculum volume. Only positive well counts between 10–100 plaques were selected for measurement. If two dilutions were within range, an average of the IU/mL calculated for each dilution was taken. TCID_50_ was calculated by the method reviewed by Staib et al. [14], and IU/mL were approximated using the Poisson distribution [23,24] as follows:

TCID_50_ = 10 − (x − d/2 + d Σr/n)

IU/mL = 0.69/(TCID_50_ × V_inoculum_)

where V_inoculum_ = inoculum volume expressed in mL; x = highest dilution giving 100% infection rate; d = log10 of dilution factor (d = 1 for a single 10-fold serial dilution [10^1^]); Σr = cumulative number of infected wells from the last dilution tested to the highest dilution giving 100% infection rate; and *n* = number of wells per dilution. See Figure A5 for schematic diagram of these techniques and calculations.

### 2.13. MVA Titer Determination by Flow Cytometry

To determine the MVA titer in the BHK-21 cells using the flow cytometry method, the protocol described in Section 2.11 was used with the following modifications: To be able to calculate IU/mL, a pre-infection cell count of the seeded BHK-21 cells was done in 4 wells using a hemocytometer on the day of infection. Media from remaining wells was removed and 100 µL of 10-fold serial dilutions of MVA virus in OptiMEM were added in quadruplicates to the different wells. After a 1 h incubation, the cells were washed and 500 µL/well complete DMEM with 0.5 µg/mL nocodazole was added. After overnight incubation, the cells were harvested and stained as described before for the extra-/intra-cellular staining, using a 3 µg/mL dilution of 33C7 or r33C7 as the primary antibodies. In all cases, the flow cytometry samples were kept in the dark at 4 °C for up to 48 h before data collection and the data was collected in a NovoCyte Quanteon 4025 (Agilent). Data analysis was performed using FlowJo v10.9, Ashland, OR, USA, following the gating strategy (shown in Figure A5) and the results were expressed as the average of quadruplicate samples. To calculate the IU/mL, obtained percent infection values were plotted against their corresponding virus dilutions to generate an infection curve, and a single point of replicate measurements within the estimated linear range of the curve was chosen for calculations, which in all cases fell below 30% infectivity. The following formula for <30% infectivity, previously described by Li et al. [16], was used:

IU/mL = (%INF × CELLS × DIL)/(100 × V_inoculum_)

where %INF = percentage of infected cells of the total live cell population (eGFP+ or Ab+ cells); CELLS = average cells/well based on the pre-infection cell count; DIL = dilution factor (DIL = 10 for a single 10-fold serial dilution [10^1^]); and V_inoculum_ = inoculum volume expressed in mL.

### 2.14. Diagrams

All diagrams describing animal treatment schedules and MVA titration protocols were prepared using Biorender.com, Toronto, ON, Canada.

### 2.15. Result Reprsentation and Statistical Analysis

All graphical representations and statistical analyses were performed using GraphPad Prism software v10.3.0, Boston, MA, USA. Differences in viral titers measured by the two different titration methods were assessed via Mann–Whitney test.

## 3. Results

### 3.1. Identification of Three New Monoclonal Antibodies against MVA Cell Surface-Binding Protein (CSBP)

**Figure A1:**
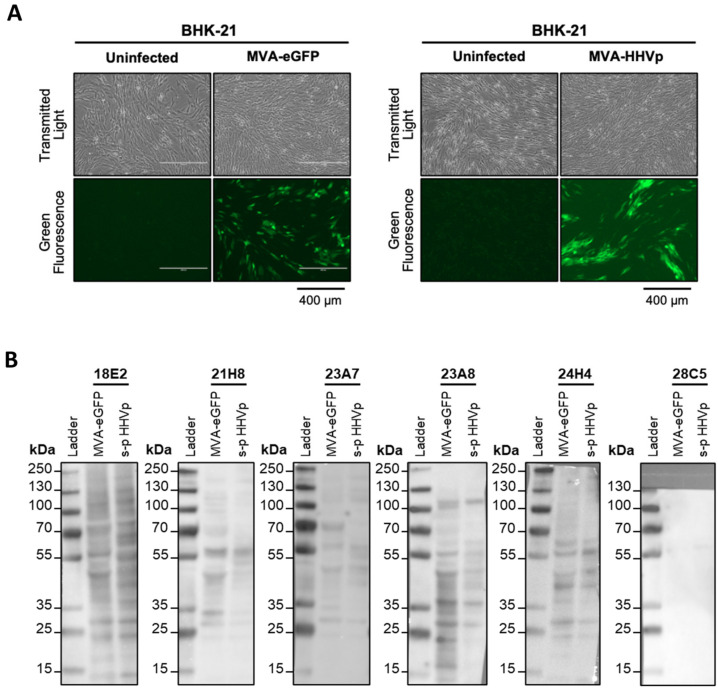
Modified vaccinia Ankara (MVA) infection of BHK-21 cells for lysate generation and human herpesvirus 4 protein (HHVp) production, and HHVp-specific antibody immunoblot assessment. (**A**) Microscopy analysis of BHK-21 cells infected with MVA-eGFP or MVA expressing His-tagged HHVp. BHK-21 cells were infected with MVA-eGFP or MVA expressing His-tagged HHVp (MVA-HHVp). Shown are representative phase (transmitted light) and GFP (green fluorescence) channel micrographs for each virus 16 h after infection; uninfected cells were used as a mock control. For generation of reagents for antibody generation and characterization, infected cells were incubated until achieving 90–100% infection based on eGFP expression as assessed by microscopy, and were harvested and processed for either lysing (MVA-eGFP-infected cells) or for protein purification (MVA-HHVp-infected cells). (**B**) Immunoblot assessment of hybridoma supernatants to detect anti-HHVp antibodies. As described in Figure 1C, culture supernatants from hybridoma clones that were ELISA-positive in Figure 1B were used as primary antibodies in immunoblot assay against lysates of MVA-eGFP-infected BHK-21 cells or semi-purified His-tagged HHVp (s-p HHVp). Shown are the blots of hybridoma clones (6/9) that did not detect a suspected MVA-specific protein band. Remaining blots (3/9), positive for suspected MVA protein, are shown in Figure 1C.

**Figure A2:**
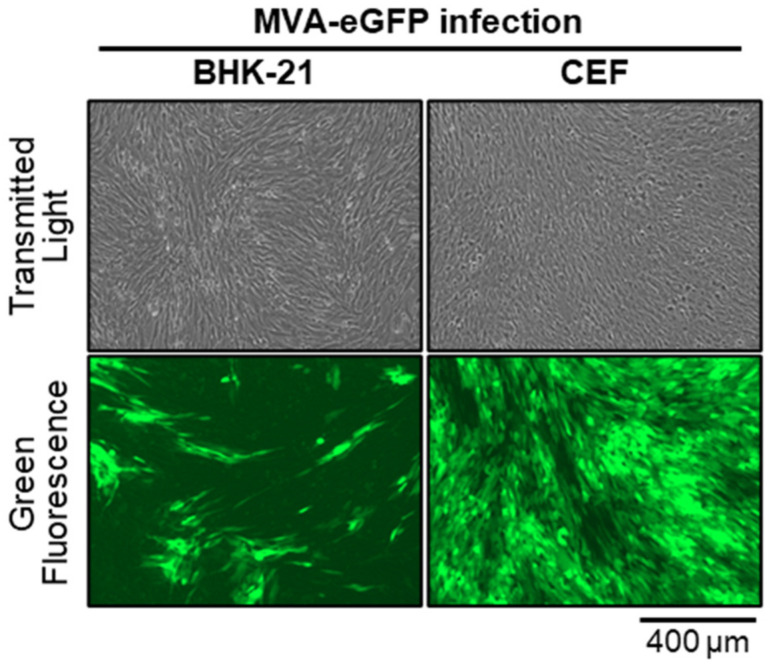
Modified vaccinia Ankara (MVA) infection of BHK-21 and CEF cells for lysate and flow cytometry sample generation. BHK-21 or CEF cells were infected with eGFP-expressing wildtype MVA (MVA-eGFP). Shown are representative phase (transmitted light) and GFP (green fluorescence) channel micrographs for each cell type 22 h after infection. For generation of reagents for antibody characterization, infected cells were incubated until achieving 90–100% infection based on eGFP expression as assessed by microscopy, and were harvested and processed for either lysing or for flow cytometry.

In an attempt to develop mAbs against a human herpes virus 4 protein, referred to in this article as HHVp, a female BALB/c mouse was immunized with three doses of an MVA virus expressing this protein (MVA-HHVp), followed by two doses of HHVp semi-purified from BHK-21 cells infected with an MVA virus expressing a His-tagged version of this protein, MVA-HHVp-His (Figure 1A). After generating hybridomas from this mouse via standard hybridoma fusion protocols [22], we conducted an initial hybridoma cell culture supernatant screening using ELISA against semi-purified HHVp-His, and we identified 21 potential HHVp-specific antibody-producing hybridomas that were further cultured until stable (week 4 post-fusion). The obtained hybridoma culture supernatants were re-screened by a confirmatory ELISA (Figure 1B), resulting in the identification of 9/21 as positive (OD_405nm_ > 0.1). These nine clones were then screened by immunoblot using semi-purified His-tagged HHVp as a target, with lysate from BHK-21 cells infected with eGFP-expressing MVA virus (MVA-eGFP) as a negative control (Figure A1A). While six clones recognized multiple bands in both samples (Figure A1B), the remaining three clones, named 9E8, 33C7, and 38D11, strongly detected a single band at approximately 37 kDa in both samples with minimal background (Figure 1C), suggesting the identification of novel MVA-specific antibodies. Given this finding, we decided to characterize the potential of these anti-MVA antibodies as tools to develop novel MVA titer quantification methods. Thus, we purified antibodies from the supernatants of the three selected hybridomas, and confirmed their purity and the presence of the heavy and light chains via SDS-PAGE and Coomassie stain analysis (Figure 1D). The specificity of the purified antibodies against MVA was further confirmed by immunoblot and flow cytometry using the MVA-eGFP-infected BHK-21 and CEF cells, and uninfected cells as a negative control (Figure A2).

**Figure 2:**
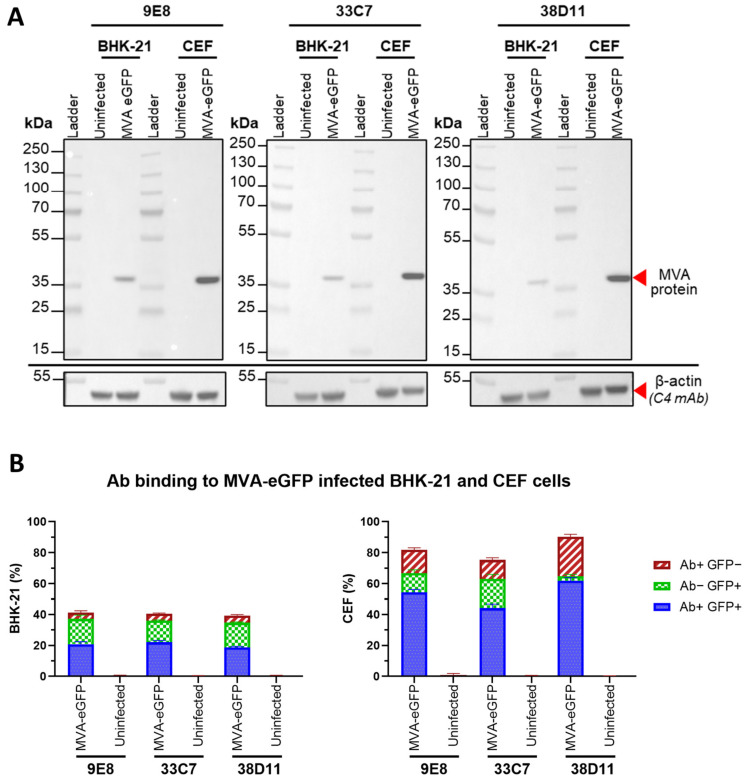
Characterization of 9E8, 33C7, and 38D11 purified antibodies against modified vaccinia Ankara (MVA) virus. (**A**) Immunoblot assessment of purified 9E8, 33C7, and 38D11 antibodies against MVA-infected cells. Purified hybridoma supernatant antibodies 9E8, 33C7, and 38D11 were used as primary antibodies in immunoblot assay against lysates of MVA-eGFP-infected BHK-21 or CEF cells. Uninfected BHK-21 and CEF cell lysates were used as negative controls. As a loading control, all samples were additionally stained with β-actin primary antibody. Expected protein sizes are indicated with red arrows. (**B**) Flow cytometry assessment of purified 9E8, 33C7, and 38D11 antibodies against MVA-infected cells. Purified hybridoma supernatant antibodies 9E8, 33C7, and 38D11 were used as primary antibodies in flow cytometry assay against BHK-21 or CEF cells infected with MVA-eGFP, using Alexa Fluor 647-conjugated anti-mouse IgG secondary antibody. Uninfected BHK-21 and CEF cells were used as negative controls. For each antibody and condition (infected versus uninfected), shown is the mean percentage (%) + standard deviation of cells from quadruplicate measurements that were positive for the antibody signal but negative for eGFP (Ab+ GFP−), negative for the antibody signal but positive for eGFP (Ab− GFP+), or positive for both the antibody signal and eGFP (Ab+ eGFP+). See also Figure A2.

Immunoblots against cell lysates of the infected cells (Figure 2A) showed that the three hybridoma clones resulted in a clean band at ~37 kDa in both samples, with no background staining present in the negative control. To ensure these results were not due to loading differences between samples, we performed β-actin staining as a loading control, and confirmed all samples were similarly loaded. For the flow cytometry analysis (Figure 2B), we carried out an extracellular staining using a high concentration of the anti-MVA antibodies (1.5 μg/sample). On the BHK-21 cells, 9E8 and 38D11 recognized ~55% of the eGFP-positive (eGFP+) cells (MVA-eGFP-infected) while 33C7 bound to ~61%, indicated by the blue bars (mAb+ GFP+); on the other hand, all three antibodies presented low binding (<5%) to eGFP-negative (eGFP-) cells (red bars). Regarding CEF cells, we found that even though 38D11 was capable of binding to most of the eGFP+ cells (~95% mAb+ GFP+), it was also bound up to 25% of eGFP- cells, indicating a high non-specificity, while 9E8 and 33C7 reached 81% and 70% of eGFP+ coverage, respectively, with a lower non-specific binding (12–15%). No antibody binding was observed in either uninfected BHK-21 or CEF cells. These data suggest that the three antibodies bind to an MVA antigen that expresses on the cellular surface of MVA-infected cells.

**Figure A3:**
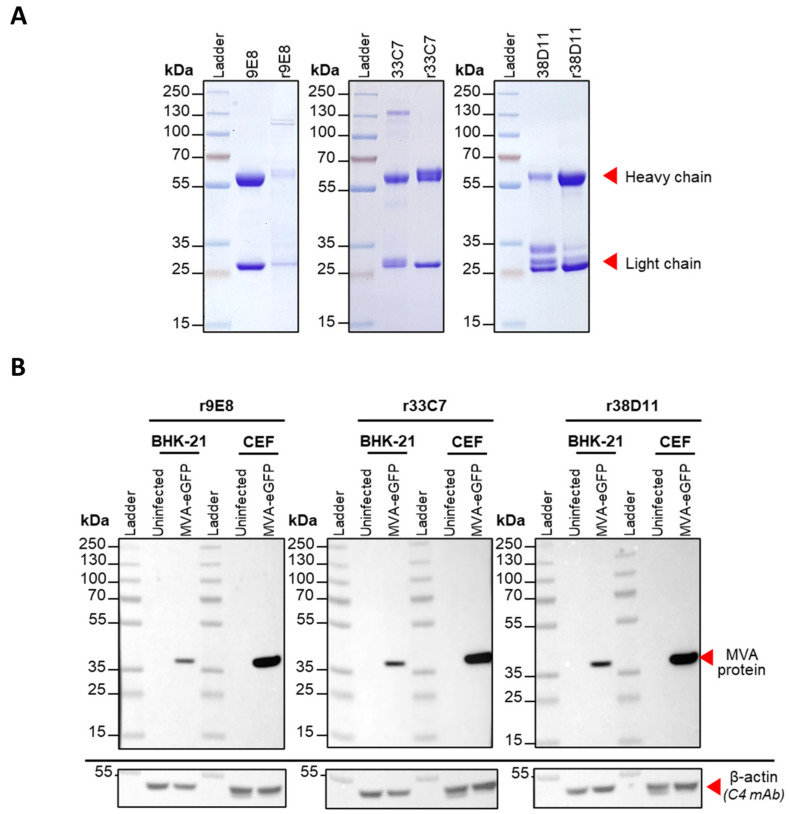
Characterization of recombinant 9E8, 33C7, and 38D11 antibodies. (**A**) SDS-PAGE analysis of purified recombinant 9E8, 33C7, and 38D11 antibodies. After 9E8, 33C7, and 38D11 hybridoma sequencing (Table 1), the CDR1, CDR2, and CDR3 sequences of each antibody were cloned into antibody expression plasmids, which were used to produce recombinant 9E8, 33C7, and 38D11 (r9E8, r33C7, and r38D11) in ExpiCHO-S cells. Recombinant antibodies were purified from the supernatants of transfected ExpiCHO-S cells via protein A (r9E8 and r33C7) or G (r38D11) affinity chromatography and subjected to SDS-PAGE followed by Coomassie Brilliant Blue staining to assess antibody purity. Heavy and light chains are indicated with red arrows. (**B**) Immunoblot assessment of purified r9E8, r33C7, and r38D11 antibodies against MVA-infected cells. Purified r9E8, r33C7, and r38D11 were used as primary antibodies in immunoblot assay against lysates of MVA-eGFP-infected BHK-21 or CEF cells. Uninfected and BHK-21 and CEF cell lysates were used as negative controls. As a loading control, all samples were additionally stained with β-actin primary antibody. Expected protein sizes are indicated with red arrows.

To confirm antibody monoclonality, we isotyped the three antibody clones and sequenced the variable region of their heavy and light chains (V_H_ and V_L_, respectively) (Table 1). Isotyping identified clones 9E8 and 33C7 as mouse IgG_1_, and clone 38D11 as mouse IgG_2a_, while the complementary–determining region (CDR) sequences demonstrated the three mAbs to be unique and distinct from each other. To verify that the obtained sequences corresponded to the anti-MVA Abs, the CDRs were subcloned into a mouse IgG1 and a mouse κ expression pcDNA 3.4 vector, respectively, and expressed as recombinant antibodies (rAbs) using an ExpiCHO™ expression system: r9E8, r33C7, and r38D11. After purification, we confirmed the presence of heavy and light chains by SDS-PAGE and Coomassie staining (Figure A3A) and the retention of their specificity against MVA by the detection of the ~37 kDa band during immunoblot assessment (Figure A3B), confirming that these antibodies are mAbs.

To identify the specific MVA antigen(s) recognized by the selected mAbs, we performed mass spectrometry analysis of the target band identified by immunoblot (Figure 2A and Figure A3B). The top five MVA specific candidates identified during two rounds of analysis are listed in Table 2 (specific mass spectrometry values from one round shown), where the two highlighted proteins, cell surface binding protein (CSBP; Accession O57211) and IMV heparin binding surface protein (IMV-HBP; Accession O57206), are the most likely to be found on the cell surface.

**Figure 3:**
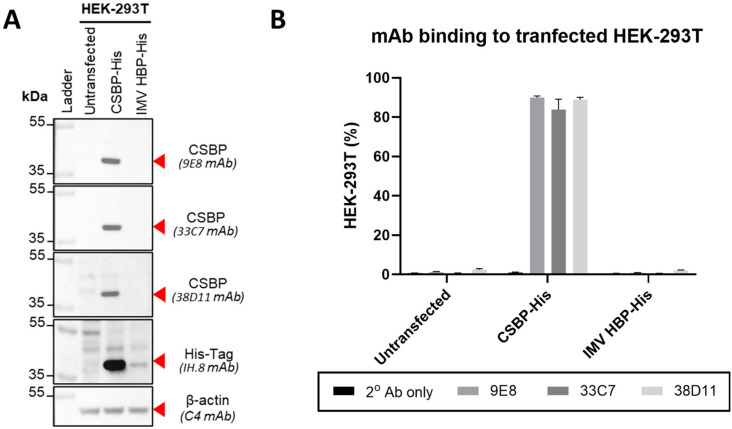
Identification of 9E8, 33C7, and 38D11 antibody targets. (**A**) Immunoblot assessment of purified antibodies 9E8, 33C7, and 38D11 against HEK-293T cells expressing modified vaccinia Ankara (MVA) proteins. Purified hybridoma supernatant antibodies 9E8, 33C7, and 38D11 were used as primary antibodies in immunoblot assay against lysates of HEK-293T cells transfected with expression plasmids coding for His-tagged cell surface-binding protein (CSBP-His) and His-tagged IMV heparin binding surface protein (IMV HBP-His), MVA proteins identified as possible antibody targets in Table 2. Un-transfected cells were used as negative controls. As a positive antibody control, samples were also processed with anti-His primary antibody. As a loading control, all samples were additionally stained with β-actin primary antibody. Expected protein sizes are indicated with red arrows. (**B**) Flow cytometry assessment of purified antibodies 9E8, 33C7, and 38D11 against HEK-293T cells expressing MVA proteins. Purified hybridoma supernatant antibodies 9E8, 33C7, and 38D11 were used as primary antibodies in flow cytometry assay against HEK-293T cells transfected with CSBP-His and IMV HBP-His expression plasmids. Un-transfected cells were used as a negative control. To control for secondary antibody background, all samples were also processed in the absence of primary antibody (2° Ab only). For each antibody and condition, shown is the mean percentage (%) + standard deviation of cells from quadruplicate measurements that were positive for the antibody signal.

Considering that both proteins were identified as top targets on two separate rounds of mass spectrometry analysis and their molecular weights are similar to the expected ~37 kDa molecular weight, we decided to clone their sequences into individual pTT3 plasmids with the addition of His-Tag as tools to test the specificity of 9E8, 33C7, and 38D11. The two plasmids were transfected into HEK-293T and the expression of CSBP and IMV-HBP was confirmed by immunoblot using an anti-His-Tag mAb (Figure 3A). The specificity of 9E8, 33C7, and 38D11 towards these proteins was then evaluated by immunoblot (Figure 3A) and flow cytometry (Figure 3B) where the three mAbs demonstrated binding to CSBP on transfected HEK-293T cells with no recognition of IMV-HBP or un-transfected HEK-293T cells, confirming CSBP to be the target of all the three mAbs.

Altogether, the results above describe the generation and characterization of three new mAbs—9E8, 33C7, and 38D11—that target the MVA CSBP protein, positioning them as a promising candidates for the development of innovative techniques to quantify MVA viral titers in infected cells.

### 3.2. Assesment of the Suitability of 9E8, 33C7, and 3D811 as Primary Antibodies for Accurate MVA Titrations by Flow Cytometry

Building on the previous success of polyclonal antibody use for MVA titration by flow cytometry [16], we aimed to refine this method using MVA-specific mAbs. To assess the suitability of 9E8, 33C7, and 38D11 for accurate MVA titration, we first optimized experimental conditions using MVA-eGFP, which allows for the tracking of infected cells via eGFP expression.

**Figure A4:**
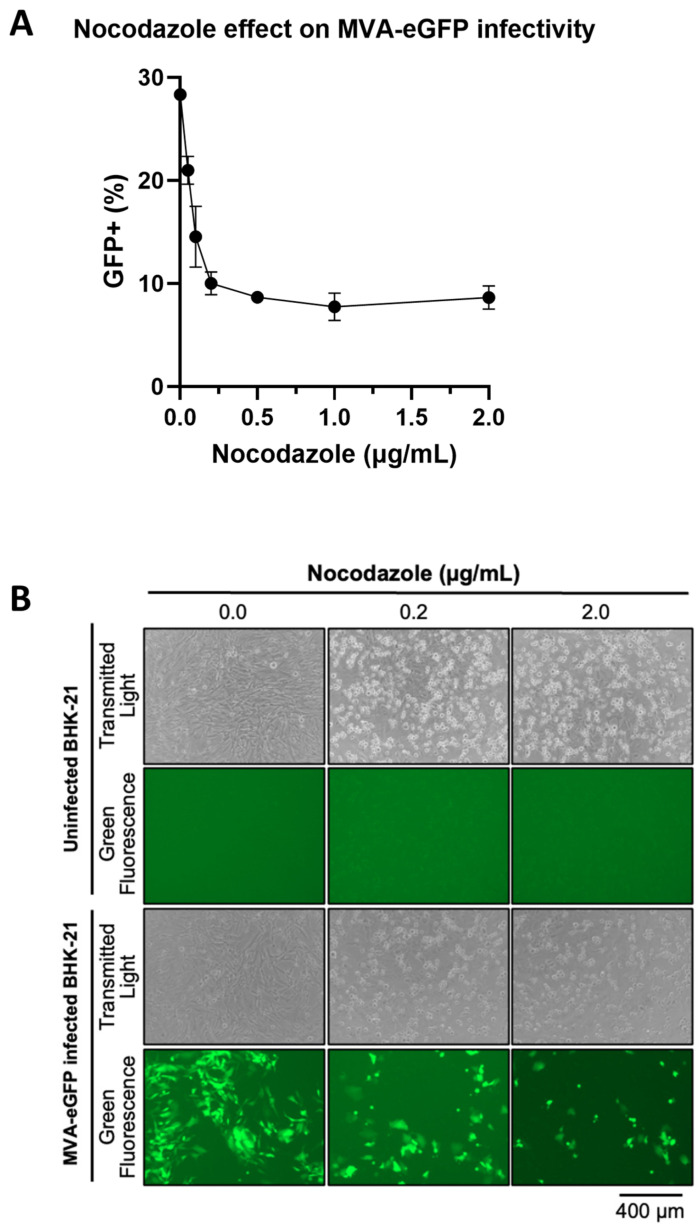
Titration of nocodazole as a viral morphogenesis inhibitor in MVA-eGFP-infected BHK-21 cells. (**A**) Flow cytometry assessment of MVA-eGFP-infected BHK-21 cells cultured in varying concentrations of nocodazole. BHK-21 cells were infected with MVA-eGFP and subsequently cultured with varying concentrations of nocodazole to inhibit viral morphogenesis and secondary infection, followed by harvesting and processing for eGFP-based flow cytometry assay. Shown is the resulting infection curve, with each dot representing the mean percentage (%) ± standard deviation of cells from triplicate infections that were positive for eGFP signal at each corresponding nocodazole culture condition. A range of 0.2–0.5 µg/mL nocodazole concentration was chosen as the optimal culture condition for modified vaccinia Ankara-infected cells for subsequent flow cytometry viral titrations. (**B**) Microscopy analysis of MVA-eGFP-infected BHK-21 cells cultured in varying concentrations of nocodazole. Shown are representative phase (transmitted light) and GFP (green fluorescence) channel micrographs of cells from (**A**) 18–20 h after infection for 0, 0.2, and 2 µg/mL nocodazole culture conditions, before harvesting; uninfected cells were used as a mock control.

Considering that an ideal viral titration should be able to quantify primary infection only [16], and that both BHK-21 and CEF cells are not only permissive but can also propagate MVA viral infection [1], we evaluated the use of nocodazole, an anti-mitotic agent, to arrest cells in the G2/M cell cycle phase, inhibit viral morphogenesis and egress, and prevent secondary infection, as previously established [16,25,26,27]. The optimal concentration of nocodazole to inhibit secondary infection without compromising cell viability was determined by an overnight culture of MVA-eGFP-infected BHK-21 cells in the presence of different concentrations of nocodazole (range of 0–2 μg/mL; Figure A4A). Uninfected cells were similarly treated to evaluate nocodazole’s cell-specific effect. Nocodazole concentrations between 0.2–2 μg/mL uniformly reached MVA-eGFP infections levels in BHK-21 cells of ~10%, while lower concentrations resulted in an increase of eGFP signal with a maximum of 30% when nocodazole was not used, suggesting a loss of secondary infection inhibition under 0.2 μg/mL. Considering this data and the increasing number of detached rounded cells at increasing nocodazole concentrations (Figure A4), we selected a range of 0.2–0.5 μg/mL of nocodazole for our subsequent experiments in BHK-21 cells. A similar experiment was performed in CEF cells; however, due to high toxicity of the nocodazole treatment in these cells, optimal nocodazole concentration to successfully inhibit secondary infection could not be determined. For this reason, only BHK-21 cells were used in the subsequent flow cytometry experiments.

**Figure 4:**
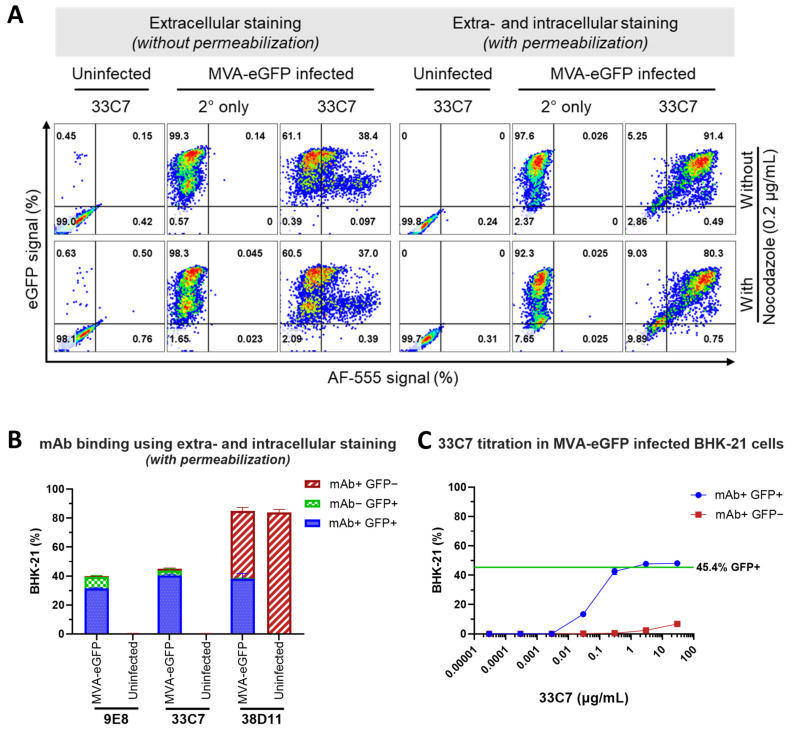
Optimization of experimental conditions for monoclonal antibody (mAb)-based flow cytometry modified vaccinia ankara (MVA) detection in MVA-eGFP-infected cells. (**A**) Comparison of 33C7-based flow cytometry assessment in non-permeabilized versus permeabilized cells infected with MVA-eGFP in the absence or presence of nocodazole. Purified 33C7 was used as primary antibody in flow cytometry assay against BHK-21 cells that were infected with MVA-eGFP under different conditions. Infected cells were either cultured in the absence or presence of nocodazole to inhibit viral morphogenesis. Following harvest, cells were processed for 33C7 staining, either without permeabilization (extracellular staining), or following permeabilization (extra- and intracellular staining). Uninfected cells were used as a negative control. To control for secondary antibody background, all samples were also processed in the absence of primary antibody (2° Ab only). Shown are representative flow cytometry plots of triplicate infections for each condition, with the *Y*-axis representing the eGFP signal and the *X*-axis representing the secondary antibody AF-555 signal. For each plot, the percentage of cells that were either positive or negative for each fluorescent signal are shown in each corresponding quadrant. (**B**) Comparison of 9E8, 33C7, and 38D11 as primary antibodies in flow cytometry-based detection of MVA in MVA-eGFP-infected cells. Purified hybridoma supernatant antibodies 9E8, 33C7, and 38D11 were used in flow cytometry assay as primary antibodies against permeabilized BHK-21 cells that were infected with MVA-eGFP in the presence of nocodazole. Uninfected cells were used as negative controls. For each antibody and condition (infected versus uninfected), shown is the mean percentage (%) + standard deviation of cells from triplicate infections that were positive for the antibody signal but negative for eGFP (mAb+ GFP−), negative for the antibody signal but positive for eGFP (mAb− GFP+), or positive for both the antibody signal and eGFP (mAb+ eGFP+). (**C**) Titration of 33C7 as primary antibody for flow cytometry detection of MVA in MVA-eGFP-infected cells. MVA-eGFP-infected BHK-21 cells cultured in nocodazole were permeabilized and processed for flow cytometry with varying concentrations of primary antibody 33C7 to identify an optimal 33C7 concentration. Unstained cells were used as a negative control. Shown are two resulting antibody titration curves, one corresponding to the population of cells that were mAb+ GFP+ and the other corresponding to the cells that mAb+ GFP−, with each dot representing the mean percentage (%) ± standard deviation of cells from triplicate infections that were positive for the antibody signal at each corresponding 33C7 concentration. The green line depicts the mean % eGFP+ cells in the unstained control set of cells. 3 µg/mL was chosen as the optimal 33C7 primary antibody concentration for staining. See also Figure A4.

Next, because 9E8, 33C7, and 38D11 mAbs target CSBP, we assessed whether nocodazole could impact CSBP expression and availability. Additionally, as a way to determine if all infected cells express CSBP in the cell surface at the evaluated harvest time, we also compared extracellular and intra-/extracellular mAb staining. To this end, we performed flow cytometry on MVA-eGFP-infected BHK-21 cells cultured with or without nocodazole (Figure 4A) and stained with 33C7 mAb using two methods: without permeabilization to assess CSBP presence on the cell surface (Figure 4A, left panel) and with permeabilization to measure intra- and extracellular CSBP (Figure 4A, right panel). While 90–100% of cells were eGFP+, indicating infection, 33C7 detected only 37–38% of the infected population (eGFP+AF-555+ cells) when an extracellular staining was used. However, with permeabilization, mAb detection increased to 90–95%, aligning with eGFP levels. No signal was observed in the secondary-only control, confirming 33C7-specific binding. Overall, nocodazole did not affect flow cytometry performance and permeabilization allowed for optimal CSBP detection, thus we proceeded with nocodazole and permeabilization for subsequent experiments.

Having optimized experimental culture and flow cytometry conditions, we next assessed the capacity of all three mAbs to detect MVA-eGFP-infected BHK-21 cells in flow cytometry at a high mAb concentration (3 μg/sample), using the optimized nocodazole culture and extra- and intracellular staining (permeabilization) conditions (Figure 4B). Results show that 9E8, 33C7, and 38D11 recognized approximately 80%, 92%, and 99% of the eGFP+ cells, respectively, indicated by the blue bars (mAb+ GFP+). In addition to displaying the highest binding to infected cells, 38D11 also presented the highest non-specific binding to eGFP− cells (red bars), reaching more than 45% detection, while 9E8 and 33C7 presented low non-specific-binding (<1.5%). Considering that it displayed high detection of MVA-eGFP infected cells and low non-specific binding, we chose 33C7 as our lead candidate and proceeded with further optimization. To reduce non-specific binding, our next step was to titrate the amount of 33C7 to be used during cellular staining through a 7-point 1/10 serial dilution starting from 30 μg/mL (Figure 4C), equivalent to the 3 μg/sample previously used. The BHK-21 cells were infected with MVA-eGFP reaching an infectivity of approximately 45%. Only at concentrations greater than 0.3 µg/mL, 33C7 could we detect most of the infection (blue line), while concentrations over 3 µg/mL were associated with an increase in the non-specific binding (red line). For these reasons, 3 µg/mL (or 0.3 µg/sample) was selected as the optimal concentration to use during MVA titration.

**Figure 5:**
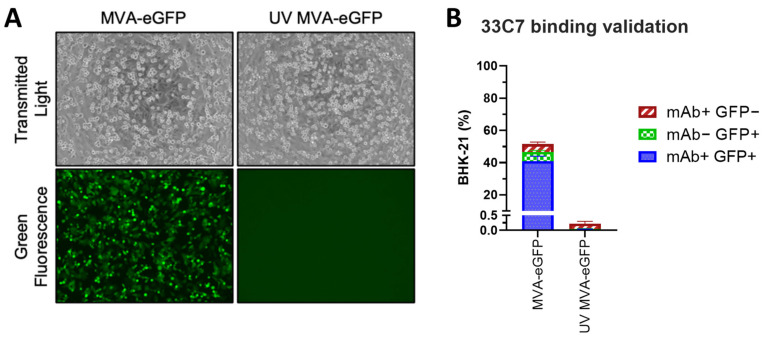
Validation of 33C7 binding to cells exposed to live versus inactivated modified vaccinia Ankara (MVA). (**A**) Microscopy analysis of BHK-21 cells infected with live or inactivated MVA-eGFP. BHK-21 cells were incubated with either MVA-eGFP (live) or UV-treated MVA-eGFP (UV MVA-eGFP; inactivated). Shown are representative phase (transmitted light) and GFP (green fluorescence) channel micrographs of cells 18 h after infection. (**B**) Confirmation of 33C7 binding specificity to live-MVA-infected cells via flow cytometry. Purified 33C7 was used in flow cytometry assay as a primary antibody against permeabilized BHK-21 cells that were incubated with either MVA-eGFP or UV MVA-eGFP in the presence of nocodazole. For each condition (MVA−eGFP− versus UV MVA-eGFP-infected), shown is the mean percentage (%) + standard deviation of cells from triplicate infections that were positive for the antibody signal but negative for eGFP (mAb+ GFP−), negative for the antibody signal but positive for eGFP (mAb− GFP+), or positive for both the antibody signal and eGFP (mAb+ eGFP+).

For viral titration, it is indispensable that the quantification is based on truly infected cells. To demonstrate that no 33C7 signal originated from the presence of viral debris or non-infectious virus particles that could be attached to the cell surface, we exposed the BHK-21 cells to either MVA-eGFP as before (live), or UV-treated MVA-eGFP (UV MVA-eGFP; inactivated). As shown in Figure 5A, no infection was observed in the UV MVA-eGFP condition, represented by a lack of eGFP signal as detected by microscopy. Subsequently, we performed flow cytometry analysis in both samples (Figure 5B), and confirmed that 33C7 binds specifically to infected cells (eGFP+).

Altogether, the data presented above highlights 33C7 as the optimal primary antibody for developing a novel flow cytometry-based method for MVA titration.

### 3.3. 33C7-Based Flow Cytometry Viral Titration Results in Higher MVA Titers than PFU, Current Standard Method

**Figure 6:**
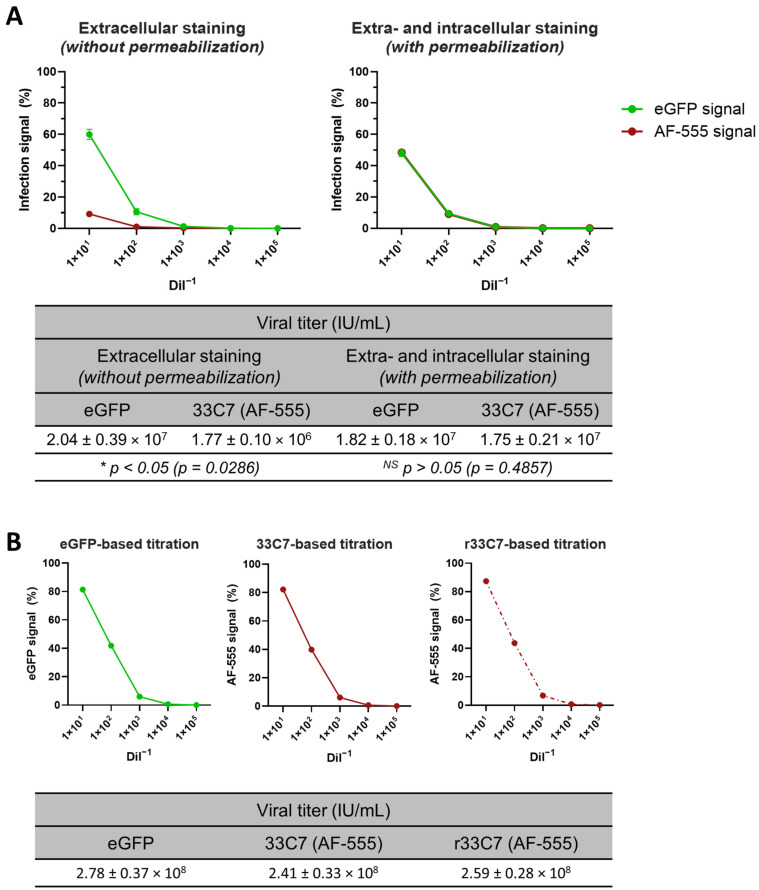
Validation of 33C7 and r33C7 as primary antibodies in flow cytometry modified vaccinia Ankara (MVA) titrations. (**A**) Comparison of eGFP- versus 33C7-based flow cytometry MVA-eGFP titer quantification in non-permeabilized versus permeabilized MVA-eGFP-infected cells. Purified 33C7 was used as primary antibody in flow cytometry assay against BHK-21 cells that were infected with varying dilutions of MVA-eGFP and cultured in the presence of nocodazole, to calculate viral titer. Following harvest, cells were processed for 33C7 staining, either without permeabilization (extracellular staining), or following permeabilization (extra- and intracellular staining). On the top row, shown are infection curves for each condition (extracellular versus extra- and intracellular staining), with each dot representing the mean percentage (%) ± standard deviation of cells from triplicate infections that were positive for eGFP or secondary antibody AF-555 signals at each corresponding virus dilution. On the bottom row, shown is a table comparing the infectious units per volume (IU/mL) calculated from each infection curve using eGFP versus AF-555 signals for each condition; each value represents the calculated mean infectious units per volume (IU/mL) ± standard deviation for each condition and signal, and the *p*-value after one-tailed Mann-Whitney test comparison of IU/mL for each signal within each condition is shown; * = significant, NS = non-significant. Permeabilization was chosen as the optimal staining condition. (**B**) Comparison of eGFP- versus 33C7- and r33C7-based flow cytometry MVA titer quantification in MVA-eGFP-infected cells under optimized experimental conditions. Purified 33C7 and r33C7 were used as primary antibody in flow cytometry assay against permeabilized MVA-eGFP-infected BHK-21 cells as in (**A**) to calculate infectious titer. On the top, shown are the resulting infection curves for each condition, where each dot represents the mean % ± standard deviation of cells from quadruplicate infections that were positive for the fluorescent signal at each corresponding virus dilution. On the bottom, a table is shown with the resulting virus titers (infectious units per mL, IU/mL) as calculated under each condition. See also Figure A5.

**Figure A5:**
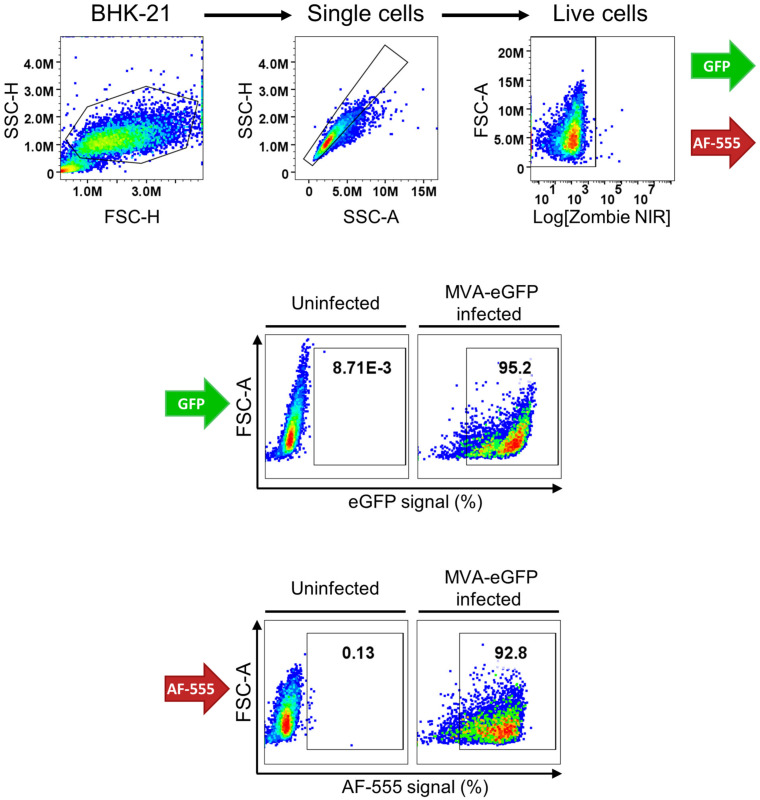
Gating strategy for 33C7-based flow cytometry modified vaccinia Ankara (MVA) titrations. Shown is the gating strategy used for 33C7-based flow cytometry for viral titration of MVA in MVA-eGFP-infected BHK-21 cells, using uninfected cells as a negative control. On the top row, representative flow cytometry plots are shown illustrating the gating of BHK-21 cells, followed by gating of single cells and live cells via a viability dye. On the middle and bottom rows, shown are the subsequent gating of infected cells based on the detected eGFP (green arrow) and secondary antibody AF-555 (red arrow) signals, respectively. For eGFP and AF-555 gates, the percentage (%) of cells that were positive for each corresponding signal is shown within each gate.

As a first proof of the use of a flow cytometry-based method for MVA titration using 33C7 as a primary antibody, we performed a titration of MVA-eGFP by infecting BHK-21 cells with 1/10 serial viral dilutions from 1/10 to 1/10^5^. After overnight incubation in the presence of nocodazole, the cells were harvested and stained using a viability dye, followed by MVA-specific staining with 33C7 at a concentration of 0.3 ug/sample and a secondary antibody conjugated to AF-555. Staining was performed with and without permeabilization to assess the impact of this treatment on viral titer determination. After flow cytometry, the data was analyzed using the gating strategy shown in Figure A5, resulting in the full titration curves and calculated viral titers presented in Figure 6A. The results without permeabilization (Figure 6A, left panel) show that 33C7 is unable to detect infection levels observed through eGFP, leading to an underestimation of the MVA-eGFP viral titer (*p* < 0.05). This discrepancy may be due to limited CSBP availability on the cell surface at the harvest time. In contrast, titration with permeabilization (Figure 6A, right panel) resulted in overlapping titration curves for eGFP and AF-555, with no differences in the calculated viral titers. Additionally, there were no differences in eGFP-based viral titers between permeabilized and non-permeabilized cells, confirming that permeabilization does not affect MVA titration outcomes. Therefore, we selected permeabilization as the optimal condition for 33C7 staining.

To verify whether recombinant 33C7 (r33C7) functions comparably to 33C7 purified from hybridoma supernatant in flow cytometry viral titration, we performed a separate titration of a different MVA-eGFP batch under the optimized conditions. In this experiment, similar titration curves and viral titers were obtained for eGFP-, 33C7-, and r33C7-based determinations (Figure 6B), demonstrating equal functionality between hybridoma-derived 33C7 and r33C7.

**Figure A6:**
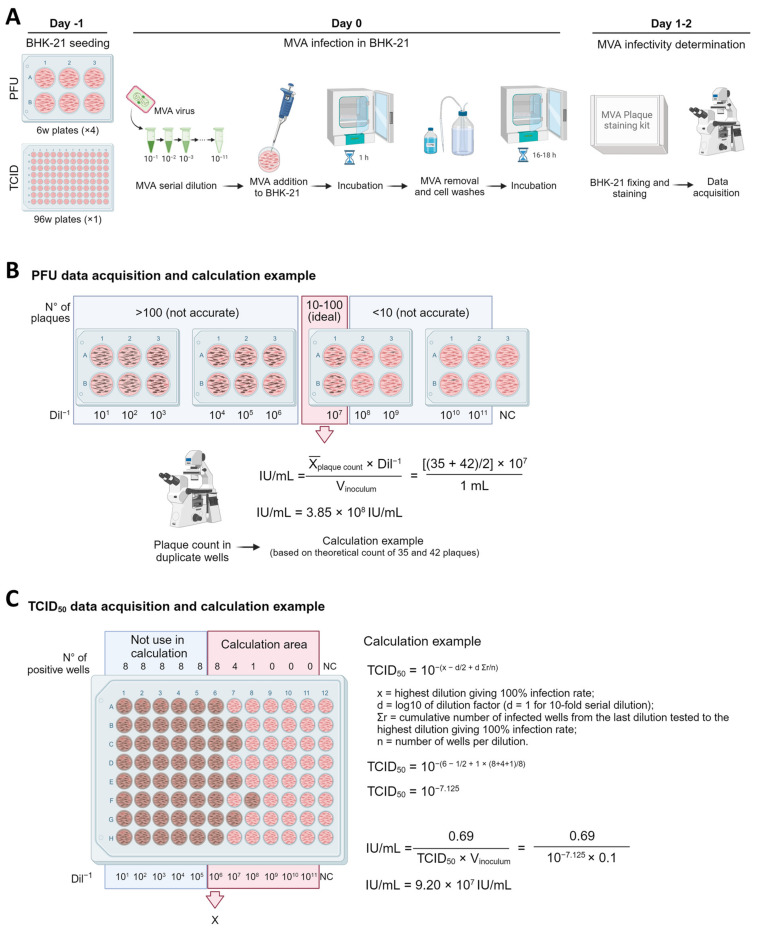
Schematic diagrams of plaque-forming unit (PFU) and median tissue culture infectious dose (TCID_50_) assays for modified vaccinia Ankara (MVA) viral titration. (**A**) Schematic diagrams of MVA titration PFU and TCID_50_ assay experimental setups. On Day-1, cells are seeded in either 6-well plates (×4) or a 96-well plate (×1) for PFU and TCID_50_ assays, respectively. On Day 0, the viral stock to be tested is serially diluted 10-fold from 10^1^ to 10^11^ and then added to the seeded cells in duplicate for PFU assay, or to each column (8 wells) of the 96-well plate for TCID_50_ assay, leaving one set of duplicates and one column uninfected, respectively, as controls. Cells are incubated for 1 h under standard cell culture conditions, then the virus is removed, and the cells are further incubated for an additional 24 h. On Day 1, the cells are fixed, and staining and data acquisition can proceed immediately or at a later time. Created in BioRender. Reidel, I. (2024) BioRender.com/h70v781 (**B**) Schematic diagram of MVA titration PFU assay data acquisition and calculation. To calculate viral titer via PFU assay, the stained plates are analyzed by microscopy. Viral plaques, which appear as dark cell clumps (brown if using DAB substrate), are counted in duplicate wells at the dilution that yields 10-100 plaques. These counts are then used in the shown formula to calculate the viral stock IU/mL. A calculation example is provided for a theoretical scenario in which 1 mL of 10^7^-diluted virus yielded 35 and 42 viral plaques in each duplicate well, respectively. Created in BioRender. Reidel, I. (2024) BioRender.com/v20y722 (**C**) Schematic diagram of MVA titration TCID_50_ assay data acquisition and calculation. To calculate viral titer via TCID_50_ assay, the fixed and stained plate is analyzed by microscopy. The last dilution that resulted in all 8 wells displaying viral plaques (i.e., 100% infectivity) and the number of wells that displayed viral plaques in all subsequent dilutions, are recorded, and then used in the shown formula to calculate the viral stock infectious units per mL (IU/mL). A calculation example is provided for a theoretical scenario in which using an infection volume of 0.1 mL, the last dilution with 100% infectivity in 8 wells was 10^6^, and the number of infected wells for the subsequent dilutions (10^7^, 10^8^, 10^9^, 10^10^, 10^11^) were 4, 1, 0, 0, and 0, respectively. Created in BioRender. Reidel, I. (2024) BioRender.com/j18g391.

Next, to validate the 33C7-based flow cytometry MVA viral titration method, we compared its performance to that of the current standard methods, PFU and TCID_50_. First, we performed a comparison between PFU and TCID_50_ by performing three independent titrations using both methods and a single batch of MVA-eGFP that was previously aliquoted to cover the six titrations. A graphical representation of the methods and calculations followed for titer determination can be found in Figure A6. The titers obtained in each titration are summarized in Table A1, where it can be observed that even though a higher average MVA titer was obtained with TCID_50_ (*p* < 0.05), this method also presented the highest variability between measurements (CV 45.54%). For this reason, we decided to adopt PFU as the reference method to validate our newly developed method.

In the first round of validation, we determined the viral titers of three MVA-eGFP batches by flow cytometry and PFU (Table 3). No differences were observed between viral titers obtained by eGFP-based or 33C7-based flow cytometry, while both resulted in higher MVA-eGFP titers than those calculated by PFU (*p* < 0.05), suggesting a higher sensitivity of the flow cytometry-based methods.

**Figure A7:**
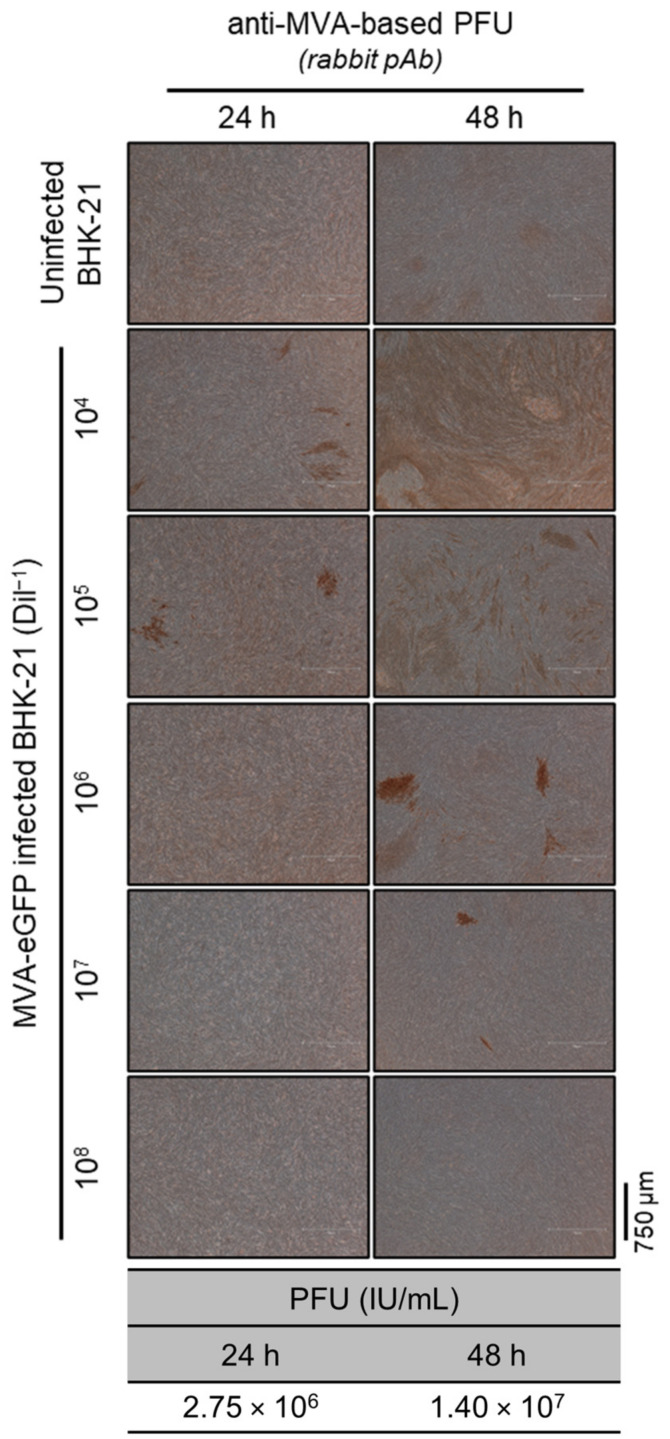
Comparison of plaque-forming unit (PFU) modified vaccinia Ankara (MVA) titration outcomes after 24 h versus 48 h virus incubations. Commercial polyclonal anti-MVA antibody was used as primary antibody for PFU assay immunostaining to titrate MVA in MVA-eGFP-infected BHK-21 cells, after either a 24 h or 48 h incubation following infection. On the top, shown are representative GFP (eGFP signal) and phase (transmitted light, antibodies) channel micrographs for selected virus dilutions under each condition after immunostaining; uninfected cells were used as a mock control. On the bottom, a table is shown with the resulting virus titers (infectious units per mL, IU/mL) as calculated under each condition.

A second validation round was done using an MVA-based EBV vaccine candidate developed by our laboratory [20], in which both eGFP and BAC genes have been removed. In this case, three independent titrations performed by independent operators through 33C7-based flow cytometry and PFU were completed using a single batch of MVA-EBV5-2 pre-aliquoted to cover the six titrations (Table 4). Higher titers of MVA-EBV5-2 were consistently obtained by 33C7-based flow cytometry vs. PFU (*p* < 0.001) with lower variability between operators (CV 36.98% vs. 51.29%, respectively). To explore whether infection/harvest time could be associated with the consistently lower MVA titers obtained by PFU assays, we performed one round of MVA-eGFP titration after 24 and 48 h of infection by this method (Figure A7). An increase in MVA-eGFP titers is observed after 48 h, with a 48 vs. 24 h titer ratio similar to ratios observed between flow cytometry vs. PFU measurements. However, it should be highlighted that PFU assays do not incorporate the use of agents to inhibit secondary infections, thus this analysis should be interpreted with caution.

Overall, the data described in this section suggests a higher sensitivity and reliability of the MVA titers obtained by flow cytometry as compared to the PFU assay.

### 3.4. 33C7 Works as a Replacement of Anti-MVA Polyclonal Serum in PFU Determinations

**Figure 7:**
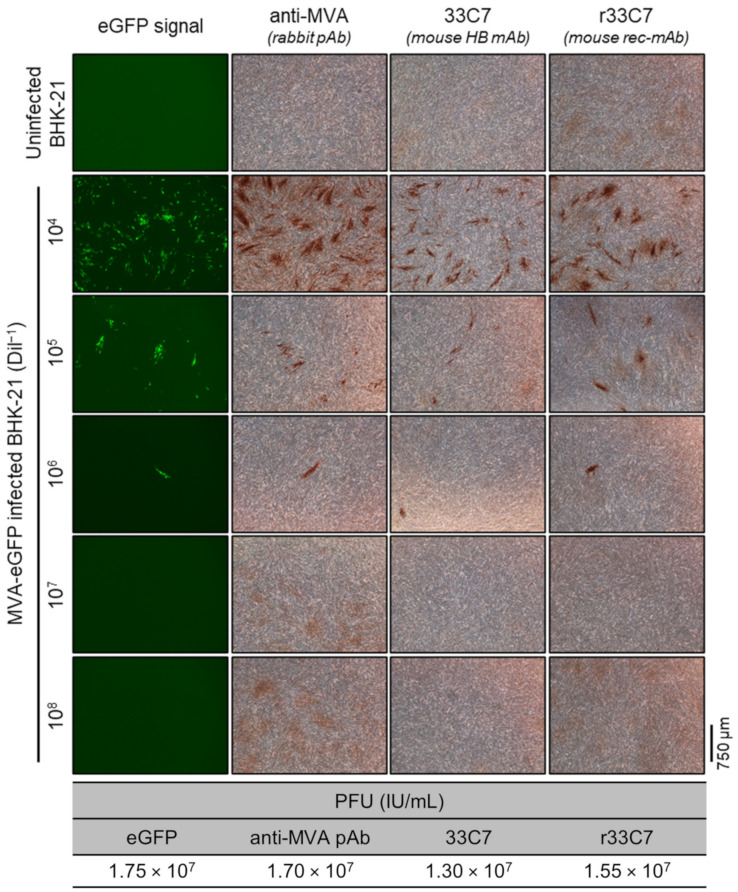
Validation of 33C7 and r33C7 as primary antibodies in plaque-forming unit (PFU) modified vaccinia Ankara (MVA) titrations. Purified 33C7 and r33C7 were used as primary antibody for PFU assay immunostaining to titrate MVA in MVA-eGFP-infected BHK-21 cells as described in Figure A6. As positive control, commercial rabbit polyclonal anti-MVA antibody was used as primary antibody in an additional set of infected cells. As an additional control, eGFP signal was also used to identify viral plaques for IU/mL calculations. On the top, shown are representative GFP (eGFP signal) and phase (transmitted light, antibodies) channel micrographs for select virus dilutions under each condition after immunostaining; uninfected cells were used as a mock control. On the bottom, a table is shown with the resulting virus titers (infectious units per mL, IU/mL) as calculated under each condition. See also Figure A6.

Recognizing that flow cytometry requires specialized equipment and that PFU remains a widely used technique, albeit limited by the current use of polyclonal antibody serum for immunostaining, we tested the performance of 33C7 and r33C7 when used for PFU determination. The results obtained from simultaneous titration of the same MVA-eGFP batch using eGFP-, anti-MVA-, 33C7-, and r33C7-based PFU methods are presented in Figure 7. All four methods yielded similar titers, however, higher background levels were observed when using the rabbit anti-MVA polyclonal serum as primary antibody. These results suggest that 33C7/r33C7 offer a promising alternative to polyclonal serum for MVA titer determination by PFU, providing a more specific and reliable option.

## 4. Discussion

The present article describes the serendipitous finding of three new anti-MVA mAbs—9E8, 33C7, and 38D11—and their capacity to bind to MVA-infected cells. This characteristic allowed us to develop a new flow cytometry-based MVA titration using 33C7/r33C7 as the primary antibodies for the detection and quantification of MVA titers with higher sensitivity and reliability than the current standard methods.

PFU and TCID_50_ assays, the current standard methods and the most widely used assays for MVA titration, are based on the formation and quantification of plaques, distinct regions of infection, in a monolayer of virus-infected adherent cells [13,14]. Plaque assays have been considered the gold standard for viral titer quantification, particularly for replication-competent lytic virions, such as the MVA parental virus vaccinia, which generates plaques that can be recognized after 48 h by staining with crystal violet or neutral red as empty spots originating from the retraction, rounding, and detachment of infected cells [13]. However, due to its high attenuation, MVA does not form clear CPE in producer cell lines, requiring a multi-step and laborious staining process to visualize the plaques [13]. Moreover, plaque assays are not conducive to simultaneous or high throughput sample processing, being a time-consuming and operator-dependent assay that requires subjective counting, which leads to results with high standard deviation [28]. Thus, as MVA gains widespread use as a viral vector for vaccine development, the need for a standardized, accurate, and reliable titration method becomes crucial to ensure precise dosing in preclinical and clinical vaccine studies [13].

Different approaches have been implemented to overcome the disadvantages of PFU and TCID_50_ titration assays over the years in the broader field of virology, leading to the development of new techniques based on the detection of viral proteins in infected cells by flow cytometry. This single-cell level approach has been successfully tested against different viruses, including influenza, adenoviruses, measles and others, demonstrating itself to be a rapid and reliable method for viral titration with an excellent capacity for high-throughput sample processing [28,29,30,31]. However, due to the nature of the measurement, this method requires a fluorescent reporter gene inside the viral genome or the availability of a specific antibody against a major viral protein [28]. While at the research level the inclusion of a reporter gene is an option that has been used for MVA pre-clinical studies [15,20], their inclusion is not acceptable for human use, requiring the use of specific antibodies for titration of viral batches to be used for this purpose. To the best of our knowledge, there is only one previous report of a method optimization for flow cytometry-based MVA quantification [16]. In this work, Li et al. used a recombinant virus, MVA-BN^®^-HER2, which encodes a fragment of the human oncogene for epidermal growth factor receptor 2 (HER-2), as the testing material, and aimed to evaluate not only the viral titer but also the expression levels of the transgene. For these purposes, the authors used an anti-vaccinia polyclonal antibody for the detection of MVA-infected cells (the IU method) and an anti-HER-2 mAb for the determination of the number of cells expressing HER-2 (the transducing units [TU] method). Their results showed that virus titers established by the IU method using permissive cells were comparable to traditional TCID_50_ titers, while the TU method can be used to provide additional information, such as the purity/genetic stability of the viral vector when the IU/TU ratio is calculated. While this first approach to the use of a flow cytometry-based method showed promising results, it shares one limitation with the current PFU and TCID_50_ assays for MVA titration: the use of a polyclonal antibody as the primary antibody for IU immunostaining.

Despite the current availability of anti-MVA polyclonal serum and its extensive use for MVA titration, it is well known that this type of antibody presents a number of disadvantages, including supply limitations and batch-to-batch variability given that they are obtained from immunized animals, as well as cross-reactivity risks, which increase with the complexity of the immunogen [32]. For example, our data shows that the use of the anti-MVA polyclonal serum in PFU assays could result in higher background levels than the use of mAbs (i.e., 33C7 or r33C7), which can represent a challenge during plaque quantification. Similarly, when undesirable antibody binding is present in flow cytometry-based methods, it translates into a high fluorescence background that can affect the measurements and therefore the final viral titer calculation [33]. Thus, considering the limitations in the use of polyclonal antibodies, and that mAbs are often preferred in applications that require regulatory approval (e.g., vaccine development) due to their high specificity and batch-to-batch homogeneity [32,34], we pursued the improvement and standardization of a general flow cytometry-based MVA titration using one of our newly characterized anti-MVA mAbs.

In this article, we started by characterizing three new anti-MVA antibodies obtained by hybridoma technology: 9E8, 33C7, and 38D11. These were selected based on their capacity to specifically detect MVA-infected cells, a required characteristic for their use as primary antibodies in viral titration assays [28]. After confirming their sequences and monoclonality, we studied their binding specificity and demonstrated that the three mAbs bind specifically to the MVA CSBP protein. CSBP is a protein conserved from the parental VACV, encoded in the MVA105L gene, also known as D8 [3]. Previous VACV studies have shown that CSBP is 304 amino-acids-long with a molecular weight of approximately 35 kDa [35], which does match the size of the target protein recognized by our mAbs in immunoblot. CSBP is a glycosaminoglycan adhesion molecule present in the viral envelope [36] and a high presence of CSBP has been found in intracellular mature virions (IMV), the first virion produced during early cellular infection [37]. These precedents have positioned CSBP as an ideal target for the detection of MVA-infected cells, and consequently the use of our anti-MVA CSBP mAbs as promising tools for MVA viral titration.

As a first stage for the development of the new flow cytometry-based MVA titration method, we optimized different experimental conditions, aiming to measure MVA primary infection only and to achieve well-distinguished populations between MVA-infected and uninfected cells. To accomplish our first goal, we evaluated the use of different concentrations of nocodazole, an anti-mitotic agent, to inhibit viral morphogenesis and the release of viral progeny [16,25,26,27]. We found that in BHK-21 cells, concentrations between 0.2–0.5 μg/mL, similar to those reported by others [16], resulted in an optimal arrest of viral release and secondary infection without significantly affecting cellular viability. However, in CEF cells, further studies are required to establish the best conditions for secondary infection inhibition. In the case of PFU assay for other viruses, physical restriction methods, such as the use of solid or semisolid overlays like agarose, methyl cellulose or carboxymethyl cellulose, are used to avoid the spread of secondary infections in cultures of 48–72 h [38]. However, due its incapacity to form clear plaques, there is a high variability in how this point is addressed for MVA, which includes the use of non-permissive cell lines such as Vero cells to avoid viral replication [39], or shorter incubation times to capture only the primary infection at the time of processing [13,14]. Both approaches present limitations: on one side, the use of non-permissive cell lines has previously shown discrepancies, as often lower viral titers are achieved when compared to permissive cell lines [40], while, on the other, the use of lower incubations times could result in the underestimation of viral titers, as we show in this article. In our experiments, we compared PFU results after 24 or 48 h incubations and we observed that after 48 h, plaques could be detected in dilutions higher than the last positive dilution observed after 24 h, indicating that these new plaques were not due to the spread of a secondary infection but a delay in the development/detection of the primary infection plaques. Taking all of this into consideration, the use of nocodazole to ensure the quantification of primary MVA infection by flow-cytometry offers an improvement over the current standard methods, representing an opportunity for the normalization of the MVA titration procedures currently used.

Towards our second goal, we aimed to increase the MVA detection without increasing the background signal. We found that the use of intracellular staining is fundamental to achieving a high detection of the infected cells, which could be explained by the intracellular localization of the IMV containing high levels of CSBP [37]. After testing the functionality of 9E8, 33C7, and 38D11 in flow cytometry using intracellular staining, we found that 33C7 presented better detectability of infected cells with lower backgrounds when used in concentrations between 0.3–3 μg/mL (30–300 ng/sample), rendering this mAb an ideal primary antibody for immunostaining. Moreover, using UV-inactivated MVA virus, we demonstrated that 33C7 only binds to truly infected cells. The use of an anti-CSBP mAb has been previously used for the assessment of VACV-infected cell levels [41], but, to our knowledge, previous studies have not used this approach for MVA titration, which makes the study presented in this article the first of its type.

After the optimization was completed, we evaluated the performance of our flow cytometry-based MVA titration as compared to PFU assay as the current standard method. Although we originally intended to compare our flow cytometry assay to both PFU and TCID_50_ assays, we found that TCID_50_ displays results with a much higher variability than PFU assay, hence we decided to focus on the latter. In a first validation, we used MVA-eGFP as the test sample, which allowed us to track true infection levels by eGFP measurement. The results showed that titration by flow-cytometry, using either eGFP or 33C7, resulted in higher MVA titers than those measured by PFU, with no difference between the eGFP and 33C7 methods, demonstrating a high sensitivity and reliability of the measurements obtained by the use of 33C7. Higher MVA titers by flow cytometry were also obtained in a second round of validation where we used the EBV vaccine candidate MVA-EBV5-2 [20] as the test article. Considering that both methods were performed after only 24 h MVA incubation, the discrepancy in MVA titers between them could be explained by the lower sensibility of PFU assays at short incubation times, as explained earlier in this article. Interestingly, we also detected that titers measured by PFU assay had higher operator variability than those obtained by flow cytometry, demonstrating the subjectivity of this method as reported by other authors [28].

Finally, we validated the use of a recombinant 33C7, r33C7, as an alternative to the use of hybridoma supernatant-purified antibody, demonstrating equivalent results. While non-recombinant mAbs depend on the survival of the parent hybridoma cell, the possibility of an unlimited supply of identical antibodies with no batch-to-batch variability offered by the recombinant mAb technology provides a great opportunity for the standardization of reliable quantification methods [32]. These characteristics, in addition to the similar performance of r33C7 vs. the widely used rabbit anti-MVA polyclonal serum, positions this recombinant antibody as a more reliable alternative even for performance of MVA PFU assays.

In summary, the data presented in this manuscript shows that it is possible to perform MVA viral titration using a mAb-based flow-cytometry method. We demonstrate that by targeting the MVA CSBP protein with a mAb it is possible to obtain accurate and reliable results in a shorter period of time than that required to acquire similar results by the PFU assay, which is the current standard method. Overall, we consider the proposed flow cytometry-based MVA titration with the use of 33C7 as a primary antibody a promising tool that should be further explored in order to pursue the worldwide normalization of MVA titration.

## Figures and Tables

**Table 1:** Anti-MVA 9E8, 33C7 and 38D11 antibody sequencing.

Anti-MVA Antibodies	Isotype	Chain	Complementarity-Determining Region (CDR) Amino Acid Sequence		
			CDR1	CDR2	CDR3
9E8	IgG1, κ	Heavy-1	DYYIH	WIDPENGDTEYAPKFQG	PGALDY
		Light-1	SATSSIIFMH	DTSKLAS	HQRNSYPWT
33C7	IgG1, κ	Heavy-1	DYEIH	GIHPGSGGTTYNQRFKG	YYGWAY
		Light-1	KASQDIYSYLS	RANNLVD	LQYDEFPFT
38D11	IgG2a, κ	Heavy-	DTYMH	RIDPANGNTKYDPKFQG	DGYYGFAY
		Light-1	SASSSVSYMH	STSNLAS	QQRSSYPFT

MVA: modified vaccinia Ankara.

**Table 2:** Mass spectrometry analysis results of potential protein target(s) bound by 9E8, 33C7 and 38D11.

Identified Vaccinia Virus Protein	Accession	Coverage%	N° of Peptides	N° of PSMs	N° of Unique Peptides	N° of Aas	MW [kDa]
**Cell surface-binding protein** **Gene = MVA105L**	**O57211**	**74**	**36**	**676**	**36**	**304**	**35.3**
Envelope phospholipase F13 Gene = F13L	A0A2I6TDP1	66	20	52	20	372	41.8
**IMV heparin binding surface protein** **GN = MVA093L**	**O57206**	**60**	**21**	**114**	**21**	**324**	**37.4**
IMV protein GN = A6L	A0A0M4R0V7	56	22	84	22	372	43.1
Telomere-binding protein I1 Gene = CVA076	A9J1L5	39	12	28	0	312	35.8

PSMs: Peptide Spectral Matches; AAs: Amino acids; MW: Molecular weight.

**Table 3:** Comparison of 33C7-based flow cytometry MVA titration to eGFP-based flow cytometry and polyclonal antibody-based PFU MVA titrations using MVA-eGFP virus.

MVA-eGFP Titers	FACS (IU/mL)		PFU (IU/mL)
	eGFP-Based	33C7-Based	
Batch 1	8.67 ± 0.58 × 10^7^	9.08 ± 0.80 × 10^7^	1.30 ± 0.14 × 10^7^
Batch 2	2.68 ± 0.35 × 10^8^	2.41 ± 0.33 × 10^8^	7.25 ± 0.92 × 10^7^
Batch 3	2.76 ± 0.73 × 10^8^	2.82 ± 0.08 × 10^8^	1.70 ± 0.14 × 10^7^
	^NS^ *p* > 0.05 (*p* = 0.9999)		
*p* value vs. PFU	* *p* = 0.05	* *p* = 0.05	

MVA: modified vaccinia Ankara; PFU: plaque-forming unit; IU: infectious unit; NS: non-significant; *: significant.

**Table 4:** Comparison of 33C7-based flow cytometry MVA titration to polyclonal antibody-based PFU MVA titration against MVA-EBV5-2 virus.

	FACS (IU/mL)	PFU (IU/mL)
	33C7	
MVA-EBV5-2 titers (IU/mL)	2.49 ± 0.12 × 10^8^	1.55 ± 0.07 × 10^7^
	1.81 ± 0.16 × 10^8^	5.15 ± 0.35 × 10^7^
	0.99 ± 0.13 × 10^8^	3.00 ± 0.71 × 10^7^
Average titers (IU/mL)	1.76 ± 0.65 × 10^8^	3.23 ± 1.65 × 10^7^
CV (%)	36.98	51.29
	*** *p* < 0.001 (*p* = 0.0001)	

MVA: modified vaccinia Ankara; IU: infectious unit; PFU: plaque-forming unit; CV: coefficient of variation; ***: significant.

## Data Availability

The raw data supporting the conclusions of this article will be made available by the authors on request.

## Appendix A

**Table A1:** Comparison of polyclonal antibody-based TCID_50_ MVA titration to polyclonal antibody-based PFU MVA titration against MVA-eGFP virus.

	TCID_50_ (IU/mL)	PFU (IU/mL)
MVA-eGFP titers (IU/mL)	1.64 × 10^7^	1.38 ± 0.04 × 10^7^
	2.18 × 10^7^	1.30 ± 0.14 × 10^7^
	3.88 × 10^7^	1.70 ± 0.14 × 10^7^
Average titers (IU/mL)	2.57 ± 1.17 × 10^7^	1.45 ± 0.21 × 10^7^
CV (%)	45.54	14.45
	* *p* < 0.05 (*p* = 0.0476)	

TCID_50_: median tissue culture infectious dose; MVA: modified vaccinia Ankara; PFU: plaque-forming unit; IU: infectious unit; CV: coefficient of variation; *: significant.
